# The Fate and Functionality of Alien tRNA Fragments in Culturing Medium and Cells of *Escherichia coli*

**DOI:** 10.3390/ijms241612960

**Published:** 2023-08-19

**Authors:** Konstantin S. Shavkunov, Natalia Yu. Markelova, Olga A. Glazunova, Nikolay P. Kolzhetsov, Valery V. Panyukov, Olga N. Ozoline

**Affiliations:** 1 Department of Functional Genomics of Prokaryotes, Institute of Cell Biophysics of the Russian Academy of Sciences, Federal Research Center Pushchino Scientific Center for Biological Research of the Russian Academy of Sciences, 142290 Pushchino, Russia; 2 Institute of Mathematical Problems of Biology RAS—The Branch of Keldysh Institute of Applied Mathematics of the Russian Academy of Sciences, 142290 Pushchino, Russia

**Keywords:** secreted RNAs, tRNA-derived regulatory RNAs, tRFs, RNA processing, circular RNAs, interspecies communications, microbial consortia, *Prevotella copri*, *Rhodospirillum rubrum*

## Abstract

Numerous observations have supported the idea that various types of noncoding RNAs, including tRNA fragments (tRFs), are involved in communications between the host and its microbial community. The possibility of using their signaling function has stimulated the study of secreted RNAs, potentially involved in the interspecies interaction of bacteria. This work aimed at identifying such RNAs and characterizing their maturation during transport. We applied an approach that allowed us to detect oligoribonucleotides secreted by *Prevotella copri* (*Segatella copri*) or *Rhodospirillum rubrum* inside *Escherichia coli* cells. Four tRFs imported by *E. coli* cells co-cultured with these bacteria were obtained via chemical synthesis, and all of them affected the growth of *E. coli*. Their successive modifications in the culture medium and recipient cells were studied by high-throughput cDNA sequencing. Instead of the expected accidental exonucleolysis, in the milieu, we observed nonrandom cleavage by endonucleases continued in recipient cells. We also found intramolecular rearrangements of synthetic oligonucleotides, which may be considered traces of intermediate RNA circular isomerization. Using custom software, we estimated the frequency of such events in transcriptomes and secretomes of *E. coli* and observed surprising reproducibility in positions of such rare events, assuming the functionality of ring isoforms or their permuted derivatives in bacteria.

## 1. Introduction

Novel types of RNAs with poorly understood functions have “emerged” over the last decades due to the activated interest of researchers in the screening of short or long non-coding transcripts in bacterial and eukaryotic cells [1,2,3,4,5,6]. Such still insufficiently studied classes of effectors are apparently far more abundant in types and numbers in all the life kingdoms than considered previously [7,8]. Their functions are diverse and, in many cases, critical for cell survival [9,10].

In higher plants and animals, they may act as regulators ensuring proper ontogenetic development [11], prevent from genomic intervention of viral agents [12,13], remodel chromatin [14,15] or otherwise affect transcription or translation processes through base pairing. In bacteria, several types of regulatory RNAs are quite extensively studied as well, including small regulatory RNAs (sRNAs) encoded by their own genes [16,17,18] and acting in *cis* antisense RNAs (aRNAs), transcribed from their own promoters located within coding sequences of target genes [19,20,21,22]. Based on the ability of sRNAs to form stable secondary structures, tools have been created for their search on a genome scale [23,24,25,26], while aRNAs can be predicted using promoter search algorithms [27]. Another type of regulatory RNA that has attracted attention in the last decades is the processed fragments of transcripts that have independent functions in cells. These include oligonucleotides cleaved from 3′-untranslated regions of mRNAs (3’UTRs) [4] and transfer RNA fragments (tRFs) [28]. These RNAs are, in most cases, short or very short [29]. So far, they cannot be predicted in silico, and special approaches are needed to elucidate the processes of their biogenesis, action and degradation.

The main subjects of our study were tRFs. They were first described in *Escherichia coli* (*E. coli*) as molecules that emerged in response to phage infection [30]. Currently, it is generally accepted that tRFs are highly abundant regulatory RNAs that are evolutionarily conserved [31]. Recent studies have confirmed the presence and functionality of tRFs in all domains of life, forming a conception that they are not random molecules resulting from tRNA degradation [32]. Though bacteria differ from higher organisms in the diversity of enzymes involved in the RNA maturation, they possess universally expressed tRNA-specific endonucleases [33]. Some tRFs are processed from long tRNAs’ precursor containing several genes. In such a case, the action of bacterial ribonucleases, such as RNases E and P, is required to yield sequences termed external transcribed spacer (ETS) and internal transcribed spacer (ITS) [34]. These spacers are usually degraded after cleavage, although the involvement of some of them in cellular regulatory events has already been demonstrated. For example, 3’ETS^LeuZ^ of glyW-cysT-leuZ polycistronic tRNA precursor base pairs with sRNAs RyhB and RybB of *E. coli* and eliminates their excess by functioning as a molecular sponge [35]. 

Mature tRNA molecules are often cleaved at the anticodon loop, providing so-called 5’ and 3’ tRNA halves [36]. These digestion products play various roles in phage infections [37] or in fighting against other bacteria [38,39]. For instance, the anticodon-loop sequence of tRNA^Arg^ is susceptible to colicin D of *E. coli*, which is an anticodon ribonuclease, and Ogawa and coauthors demonstrated that the cleaved tRNA^Arg^ halves occupy the ribosome A-site, leading to the impairment of translation [39]. Smaller stable tRFs 14–30 nt in length can also be produced from mature tRNAs by endo- and exonucleolysis [40]. In eukaryotes, fragments of the 5′- and 3′-ends of tRNAs (5′tRFs and 3′tRFs, respectively) are usually cut by Dicer nuclease near the D- or Ψ-loop of tRNAs [32,41]. The existence of a specific enzyme machinery for bacterial tRF biogenesis is still hypothetical. However, it is known that tRFs are actively exported by extracellular vesicles [42,43], which deliver them to eukaryotic cells [43,44], where they promote a variety of cellular responses [45].

The available information on RNA-mediated interspecies interactions in bacterial communities is very limited, but it is intuitively clear that microbial secretomes may be the main RNA sources involved in such communications. Their investigation began quite recently [42,43,46,47], and all extracellular RNAs studied to date were able to penetrate into cells of both their own and other species [47], but the efficiency and specificity of this internalization have not yet been quantitatively characterized. Extracellular milieu contains many RNases, but RNAs there were found to be stable not only due to their secretion in membrane vesicles [48,49,50] but also due to their protection by RNA-binding proteins [51], which have not yet been identified in bacteria.

If secreted RNAs are involved in intercellular communications, then, in recipient cells, they should be able to influence the implementation of the genetic program. So far, there is very little evidence of this affection. There are several examples indicating that co-cultivation of two different bacterial species affects gene expression in one or both organisms [46,47,52,53], but not all changes may be caused by RNA-mediated regulation. Hermansen et al. [54] studied the interactome of nasal isolates of *Staphylococcus aureus* and *Staphylococcus epidermidis* under conditions of individual and spatially separated growth of two clones on an agar plate. In both species, the expression of several virulence genes was detected, as well as transcription of a *S. epidermidis* aRNA to serine protease ESP mRNA, which was specifically suppressed in the presence of *S. aureus*. Another example is the individual and joint cultivation of bacteria that are in the predator–prey relationship (*Myxococcus xanthus*–*E. coli*). Surprisingly, *M. xanthus* had virtually no transcriptional response to the presence of prey, although hundreds of genes were differently expressed by prey in response to a predator attack [55,56]. This is all the more surprising since hundreds of *M. xanthus* genes changed their expression level when killed rather than alive *E. coli* cells were added to their population.

Methodologically significant in understanding the potential mechanisms underlying the action of secreted/imported transcripts are the results obtained by Lybecker et al. [57], who found that RNA-RNA duplexes in *E. coli* cells are cleaved by RNase III. This means that the lack of RNA-interference systems involving Ago nucleases (RISC complexes) in most bacteria does not exclude the targeted destruction of cellular transcripts using endogenous or exogenous antisense RNAs that has already been implemented in “programmable RNA antibiotics” designed to target key cellular mRNAs [19,58]. This opens a way to not only kill certain bacteria but also fine-tune microbiomes by posing bacteriostatic pressure on some taxa. Further progress in this area requires more information about the RNAs naturally involved in bacterial communications. Guided by the idea that secreted oligonucleotides are evolutionarily adapted to maintain the homeostasis in interspecies interaction, we considered the search for such candidates among tRFs and characterizing their maturation during transport as the main objectives of the study.

## 2. Results

### 2.1. Assessing Interspecies Relations of E. coli with Prevotella copri (P. copri) and Rhodospirillum rubrum (R. rubrum)

Based on the positive correlation between the abundance of bacteria belonging to the genera *Prevotella* and *Rhodospirillum* in the human intestine, it is assumed that they are in a symbiotic relationship, while the presence of both of these taxa negatively correlated with *Escherichia* [59]. Assuming that these genera exploit evolutionarily established relationships with the participation of signaling RNAs, we have already used this combination of bacteria previously [47], and implementing a differential analysis of common secretomes in mixed populations (*E. coli*–*P. copri* and *E. coli*–*R. rubrum*) showed that the presence of cohabiting bacteria altered the set of RNAs secreted by *E. coli*. In this study, we tested the ability of cell-free culture media with secretomes of *P. copri* and *R. rubrum* to affect the growth of *E. coli*.

**Figure 1:**
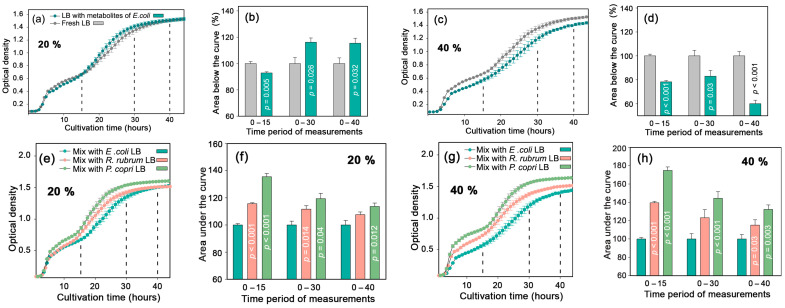
In binary bacterial consortia, *P. copri* and *R. rubrum* stimulated the growth of *E. coli*. The graphs represent the effect from the addition of 20 or 40% LB conditioned by *E. coli* (**a**–**d**), *P. copri* and *R. rubrum* (**e**–**h**). Bacterial growth was tracked by the change in OD_650_ in different time intervals, using the areas under the dynamic curves as a sensitive integral parameter and GraphPad Prizm 5 software [60] for their calculation. Student’s *t*-test based on 5 (**b**,**f**) and 3 (**d**,**h**) independent biological experiments was applied for statistical assessment.

Dilution of fresh LB with 20% nutrient-depleted broth from *E. coli* culture caused an expected but very weak suppression of *E. coli* growth only over the first 15 h of cultivation (Figure 1a,b). In the second logarithmic phase, reflecting metabolic adaptation to a less preferred carbon source, bacteria in the mixed medium grew faster than control cells cultured in fresh LB (gray plots and bars) but reached the same plateau by 40 h. When 40% of fresh LB was replaced with already used broth, the expected suppression of *E. coli* growth was observed (Figure 1c,d). However, when similar portions of broth taken from cultures of *P. copri* (green plots) or *R. rubrum* (magenta plots) were added to *E. coli* cells, only a stimulating effect was observed, compared to the growth of *E. coli* in a mixed broth with its own metabolites (Figure 1e–h). Thus, it became clear that *E. coli* senses the presence of metabolites secreted by *P. copri* and *R. rubrum*, and in simple mixed populations, their influence turned out to be positive.

### 2.2. Search for RNAs Secreted by P. copri or R. rubrum with a Potential Effect on E. coli

**Figure 2:**
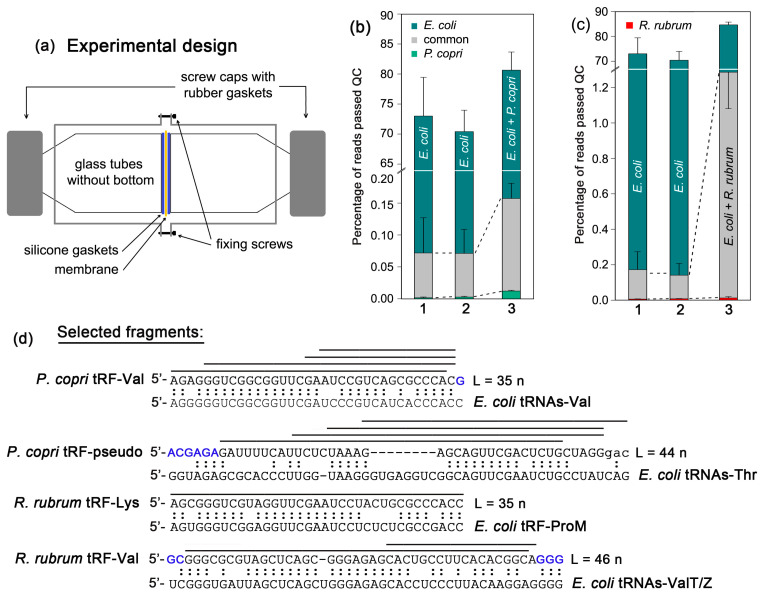
Selection of RNAs secreted by *R. rubrum* and *P. copri* and imported by *E. coli*. (**a**) Chamber designed for co-cultivation of bacteria in individual compartments. (**b**,**c**) Mapping efficiency of 16–50 nt long reads found inside *E. coli* cells on the genomes of *E. coli*, *P. copri* and *R. rubrum*. Individual growth of *E. coli* was carried out either in M9 medium (bar 1 of (**b**,**c**)) or in LB broth (bar 2 of (**b**,**c**)). Co-culturing with *P. copri* or *R. rubrum* (bar 3 of (**b**,**c**)) was performed in LB medium. In all datasets, reads were classified into three categories: Those belonging only to *E. coli* (cyan parts of the plots), “common” sequences fitting to the genomes of *E. coli* and other bacteria (gray parts) and reads unmapped to the *E. coli* genome but matching to the *P. copri* (green) or *R. rubrum* (red) genomes. (**d**) Alignment of the tRNA fragments (tRFs) selected for analysis (upper case letters) to the sequences of their closest homologues in *E. coli*. Extensions made in synthetic oligos (see below) are marked in blue. Horizontal lines denote oligonucleotides found in *E. coli* cells.

Assuming that RNAs secreted by *P. copri* or *R. rubrum* contribute to the observed interspecies communications, we designed a chamber that was composed of two compartments separated by a membrane impermeable for bacteria (Figure 2a). We then searched for *R. rubrum* and *P. copri* RNAs imported by *E. coli* after its co-cultivation with those bacteria in separate compartments, RNA isolation and sequencing. To identify internalized RNAs, 16–50 nt long reads (Eco_Pr_1/2 and Eco_Rh_1/2 sets in Supplementary Table S1) were mapped to the genomes of *E. coli* and co-cultured bacteria. Since bacteria have many orthologous genes and predominantly secrete short RNAs, some of them were mapped to both genomes even in monocultures (bar 2 in Figure 2b,c). In *E. coli*, their percentage was higher for *R. rubrum* RNA products than for transcripts of *P. copri* (gray parts of bars), which corresponds to a closer phylogenetic relationship of two *Proteobacteria* (*E. coli* and *R. rubrum*) compared to *P. copri* belonging to *Bacteroidetes*. The percentage of reads that mapped only to the *E. coli* genome was at least 500 times higher (cyan parts of bars), but there was also a small portion (0.005 ± 0.001 and 0.002 ± 0.001% for *R. rubrum* and *P. copri*, respectively) that map only to the alien genomes (green and red parts of bars). Since the transcriptomes of cells grown in M9 medium have approximately the same ectopic presence (bar 1 in Figure 2b,c), this cannot be explained by contamination from LB broth. Sequencing errors and single nucleotide polymorphisms (SNPs) in bacterial populations contribute to this background, leading to the appearance of the main part of “foreign reads” with only one mismatch to the genome of *E. coli*.

Under conditions of co-cultivation (bar 3 in Figure 2b,c), the percentage of intracellular reads matching only to the genomes of *P. copri* or *R. rubrum* increased up to 0.012 ± 0.001 and 0.01 ± 0.007%, respectively. Although the main changes occur in the number of common sequences (gray parts in bar 3), we selected the tRNA fragments (tRFs) for further analysis from 217 types of oligonucleotides matching only to the genome of *P. copri* and 56 RNAs fitting to the genome of *R. rubrum*. When all reads with one mismatch to the *E. coli* genome were removed, which reduced the background given by SNPs and sequencing errors, we obtained 198 RNA fragments with a high probability of being secreted by *P. copri* and only 28 oligonucleotides, which may be of *R. rubrum* origin.

Both sets mainly consisted of rRNA and tRNA fragments with an average size of 29 nt. They vary in the ability to form secondary structures, which can be deemed to be important for intercellular transport, and they also possess different numbers of discriminative nucleotides, which are needed for the identification of model RNAs in transcriptomes and secretomes. Based on these criteria, we chose fragments of four tRNAs (Figure 2d). They are parts of naturally structured molecules and are also able to form stem–loop elements. All the selected tRFs have multiple deviations from the nearest homologous sequences in *E. coli* transcripts (Figure 2d). tRF-Val from *P. copri* (tRF-Val_Pc_) and tRF-Lys from *R. rubrum* include the Ψ-loop and are homologous to the 3′-ends of five valine and one proline tRNAs of *E. coli*, respectively. The *R. rubrum* valine tRNA fragment (tRF-Val_Rr_), on the contrary, originated from the 5′-end. It includes the D-arm with the anticodon stem–loop structure and is homologous to the first half of *E. coli* ValT and ValZ tRNAs. The tRF-pseudo from *P. copri* is homologous to two threonine tRNAs of *E. coli*.

### 2.3. Assessing Fragmentation of Selected RNAs in the Medium of Mixed Populations

**Figure 3:**
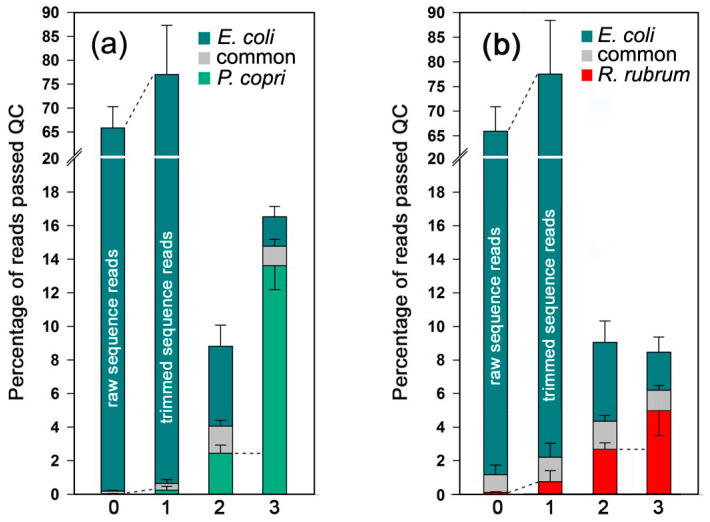
*E. coli* RNA secretome size depends on the presence of *P. copri* and *R. rubrum.* (**a**,**b**) Mapping of milieu-derived raw (bar 0 in (**a**,**b**)) and trimmed (bars 1–3 in (**a**,**b**)) 16–50 nt reads on the genomes of *E. coli*, *P. copri* and *R. rubrum*. The design of the experiment with biological samples (0–3) used for analysis is presented in the Materials and Methods section. Individual growth of *E. coli* was carried out either in M9 medium (bars 0 and 1 of (**a**,**b**)) or in LB broth (bar 2 of (**a**,**b**)). Co-culturing with *P. copri* or *R. rubrum* (bar 3 of (**a**,**b**)) was performed in LB medium. The reads were sorted into the same three categories as described in Figure 2b,c.

The objectives of this part of the study were to assess the presence of selected tRFs in the secretomes of *P. copri* and *R. rubrum* grown in mixed populations with *E. coli*, to determine the type of their fragmentation in the culturing medium and to estimate the relative amount of RNAs secreted by different bacteria. For this we used seven datasets (Eco_Prev_1-4 and Eco_Rhod_1-3, Supplementary Table S1) obtained previously [47]. In order to improve the poor mapping efficiency of secreted RNAs, the reads were trimmed from both ends. Removal of 1 nt from the 5′-ends and 3 nt from the 3′-ends, which eliminates ligation errors and extensions resulting from polyadenylation of bacterial RNAs, increased the percentage of mapped reads by about 10% even for samples from M9 medium (bars 0 and 1 in Figure 3a,b).

For secretomes, this gave on average 2.2% and about 6.5% of precisely mapped reads from monocultures of *E. coli* (Eco_out_LB_1-3). The ectopic presence of alien sequences in LB medium was at the level of about 2% (bar 2 in Figure 3a,b), which was higher compared to the samples collected from M9 medium (bar 1 in Figure 3a,b). Considering this value as a background level, the mixed population *E. coli*–*R. rubrum* contained secretomes that were approximately equal in size (bar 3 in Figure 3b), but the *P. copri* secretome was at least 6 times larger (bar 3 in Figure 3a), thus explaining the higher number of *P. copri* RNAs in datasets with oligos imported by *E. coli*. The presence of cohabiting bacteria in both cases reduced the percentage of external *E. coli* RNAs that, again, was more pronounced in the pair *E. coli*–*P. copri* and may be associated with a higher effect of the *P. copri* culture medium on the growth of *E. coli* (Figure 1e–h).

To collect derivatives of selected tRFs in *P. copri* and *R. rubrum* secretomes, four datasets obtained in mixed populations of *E. coli*–*P. copri* (Eco_ Prev_1-4, Supplementary Table S1) and three sequence libraries from the *E. coli*–*R. rubrum* pair (Eco_Rhod_1-3) were pooled. Reads that mapped to the *E. coli* genome with one mismatch were removed, and unique 12-mers present in the context of chosen tRNA fragments but absent in the *E. coli* genome were used as markers of corresponding reads. 

**Figure 4:**
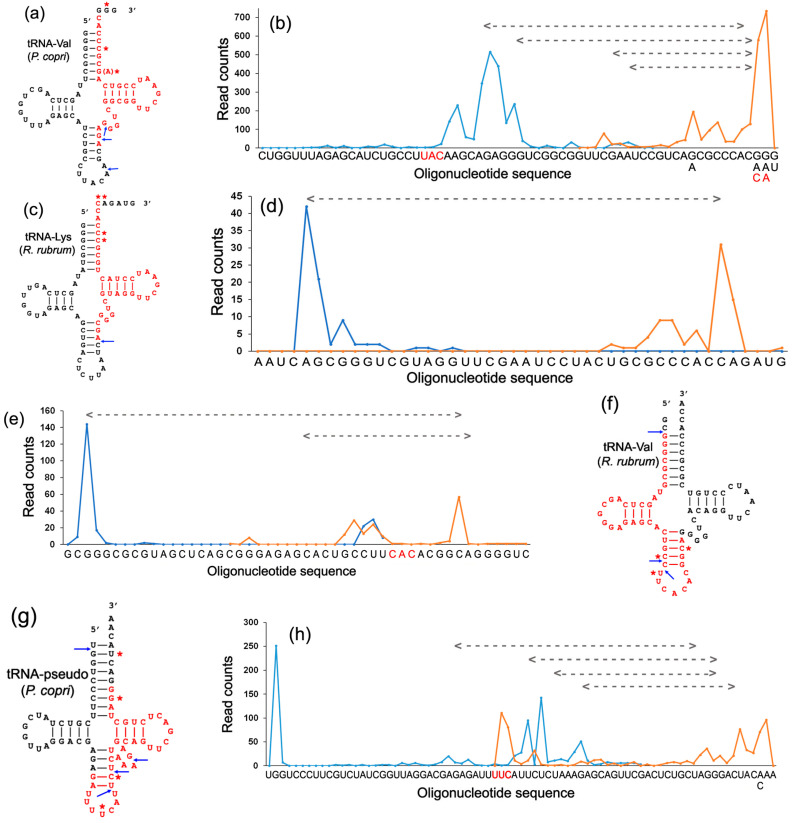
Secretomes of *P. copri* and *R. rubrum* contain tRFs found inside *E. coli* cells. (**a**,**c**,**f**,**g**) Location of the fragments imported by *E. coli* in the tRNA structure (red letters). (**b**,**d**,**e**,**h**) Distribution of 5′- and 3′-ends of reads (blue and orange plots, respectively) along the sequences of target tRNAs. The positions of the ends, corresponding to the main peaks, are marked with arrows and asterisks on the structural models (**a**,**c**,**f**,**g**). Dashed lines show oligos found in *E. coli* cells. Anticodons, if present, are printed in red. The letters below the sequences in (**b**,**h**) indicate local polymorphism.

Fragments of all model tRNAs were found in abundance in all mixed secretomes and aligned using the Clustal Omega multiple-sequence alignment tool on the EMBL-EBI portal. The positioning of their 5′- and 3′-ends was determined based on this alignment (Figure 4b,d,e,h), providing four main observations: (1) All model tRNAs were subjected to endonuclease cleavage, predominantly producing fragments with fixed 5′-ends. (2) This cleavage usually takes place in the stem of the anticodon arm either upstream (tRNA-Val of *R. rubrum*) or downstream (panels a, c, f and g) of the anticodon triplet. (3) Nearly all reads of tRNA-Val of *R. rubrum* and tRNA-pseudo were truncated for 1 nt at the 5′-end (panels e–h). (4) Fragments corresponding to the 3′-ends of tRNAs were more diverse (panels a–d, g and h) but also demonstrated predominant sites of intramolecular cleavage (panels e–h). In the case of *P. copri* valine tRNAs, which are produced from two paralogous genes varying in the sequences at the 3′-ends of the precursors (…ACGGG and …ACAAT), only processed fragments with the acceptor CCA were found (marked in red in Figure 4b).

Thus, even in the extracellular fraction, the majority of model tRNA fragments is not just a mixture of randomly digested oligonucleotides. The presence of molecules with identical ends and their large number suggested a certain functionality, which was verified experimentally using synthetic analogs of selected tRFs. With the hope of tracing the expected nucleolysis, the sequences of tRFs-Val were extended for 1-3 nt at the 5′- and/or 3′-ends, and 6 nt was added to the 5′-end of tRF-pseudo (Figure 2d).

### 2.4. Dual Effects Exerted by Synthetic Analogs of Model RNAs on the Growth of E. coli

**Figure 5:**
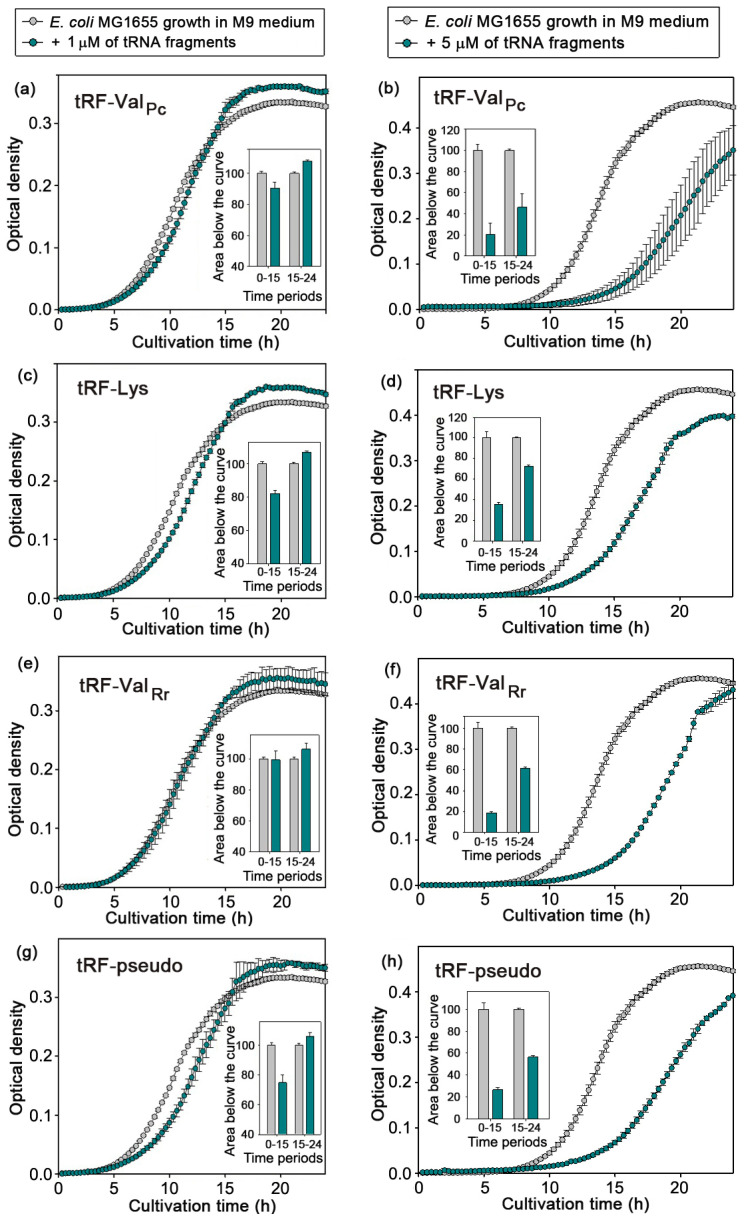
Examples of dynamic curves demonstrating the phase-dependent duality of the biological action of model RNAs at lower (panels **a**,**c**,**e**,**g**) and higher dosages (panels **b**,**d**,**f**,**h**). Cells were cultured 24 h in plate wells of a multimode reader in M9 medium with 0.5% glucose (5% N_2_ and CO_2_). The dynamics of their growth was characterized by the changes in OD_650_ over two time periods (0–15 and 15–24 h), using the areas under the plots of experimental and control samples. Statistics shown by bar plots are based on at least 3 independent biological trials.

Four oligos (Figure 2d) were chemically synthesized and used to evaluate their influence on the growth of *E. coli*. At concentrations 0.5–1 µM, all of them slightly increased the density of the cell population during steady growth (Figure 5a,c,e,g). This was in line with the observed stimulatory effect of “stationary phase” broth obtained from *P. copri* or *R. rubrum* cultures and added to the *E. coli* broth (Figure 1e–h). Although in three cases (Figure 5a,c,g) the outstripping growth of the experimental samples was preceded by some suppression, the stimulatory effect itself could be due to the mere contribution of the synthetic RNAs added to the bacteria nutritional source. Therefore, the growth experiments were repeated with higher concentrations of synthetic oligos (5 μM), which allowed us to reveal their obvious bacteriostatic effect (Figure 5b,d,f,h).

Thus, it became clear that *E. coli* senses the presence of alien tRFs and responds to them in a dose-dependent manner. The inhibitory effect observed at high concentrations is most likely due to base pairing of model tRFs with cellular transcripts, followed by their cutting by RNAse III [57]. The conformity of the stimulatory effect observed for all model tRFs is more interesting and will be the subject of a special study.

### 2.5. Fragmentation of Synthetic Analogs of Model tRFs in Culture Medium

The availability of functionally significant synthetic analogs of model tRFs made it possible to detect their processing in the culture medium to quantify the efficiency of their penetration into cells and to trace the modifications that occur during import. We, therefore, added all the four synthetic analogs to the *E. coli* culture to a final concentration of 1 µM each and analyzed their post-culturing fate via RNA-seq.

**Figure 6:**
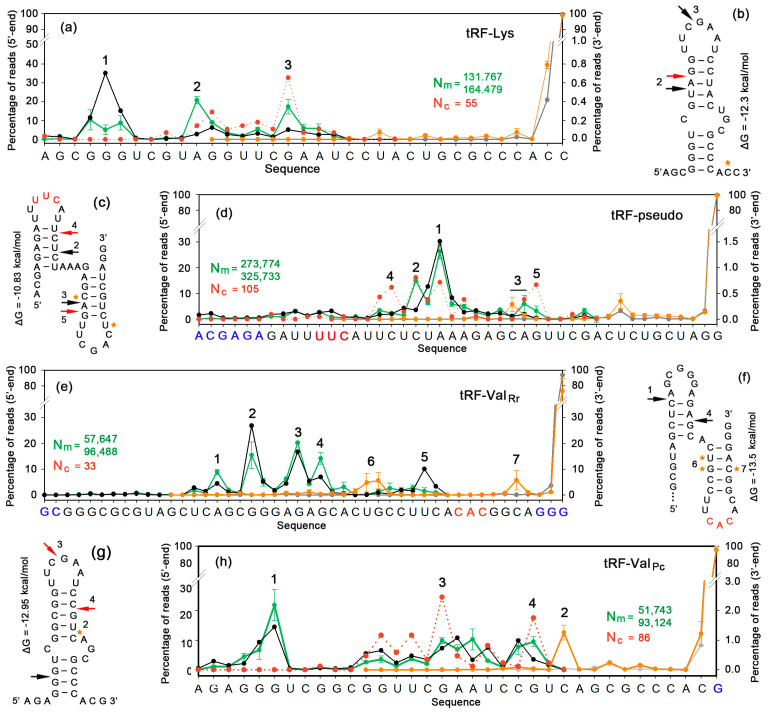
Synthetic analogs of model tRFs are cleaved by nucleases in the culture medium of growing *E. coli*, and their fragments penetrate into the cells. (**a**,**d**,**e**,**h**) The distribution of the 5′- and 3′-ends of synthetic analogs (black and gray plots, respectively) and culture media-derived sequences (green and orange plots, respectively). The dotted plots show the distribution of 5′-ends for reads detected within *E. coli* cells. N_m_ is the number of tRFs found in milieu from 2 experiments. N_c_ is the number of such reads found in pooled datasets received in the same experiments inside bacterial cells. (**b**,**c**,**f**,**g**) Structural models of tRFs. Arrows indicate preferred nuclease cleavage sites generating 5′-ends in culture medium (black) and within recipient cells (red). Asterisks show 3′-terminal nucleotides of reads predominantly accumulated in the milieu.

The obtained four sets of reads from bacterial cells (Eco_in_oligos_1/2 in Supplementary Table S1) and separately from acellular culture medium (Eco_out_oligos_1/2) were compared to synthetic oligos sequenced separately (synthetic oligos in Supplementary Table S1 and Figure 6) in terms of the distribution of their 5′- and 3′-ends.

The 3′-ends were virtually standard in all samples (gray plots in Figure 6), which is due to the 3′ → 5′ direction of chemical synthesis. A high number of obtained fragments made it possible to detect even rare exonucleolysis events at this end. As a result, an increased number of reads with penultimate cytidines at the 3′-end was found for tRF-Lys and tRF-Val_Pc_ (orange and gray plots in (Figure 6a,h). It was low (about 0.4%) and was not accompanied by further truncation. The 3′-end of tRF-pseudo was stable (orange and gray plots in (Figure 6d), but the number of untruncated reads in the tRF-Val_Rr_ set decreased by more than 20% (orange and gray plots in Figure 6e). An enhanced endonuclease cleavage in the stem of the anticodon arm (peaks 6 and 7 in panel e) and decreased number of reads lacking the terminal G from 3.62% to 0.932 ± 0.003% contribute to the overall difference.

For the 5′-ends, the situation was completely different. Although premature termination of chemical synthesis caused size heterogeneity in all samples (black plots in Figure 6), we still revealed well-pronounced and reproducible endonuclease cleavage generating new 5′-ends. In the case of tRF-Lys, the main fraction of synthetic products was only 4 nt shorter than required (peak 1 in Figure 6a). Incubation with *E. coli* cells in both experiments caused the same size redistribution in the pool of extracellular tRF-Lys with the formation of peaks 2 and 3 in the 5′-end positioning profile (green plots in Figure 6a). From the alignment of the obtained reads, it became clear that most of the RNAs from the dominant peak of synthetic molecules underwent endonucleolysis at the sites marked by black arrows on the secondary structure of tRF-Lys (Figure 6b). The milieu may, therefore, contain endonucleases that reproducibly process synthetic analogs of alien RNAs.

The tRF-pseudo profile (Figure 6d) exemplifies another type of the same phenomenon. Synthetic analogs were more homogenous, showing only one dominant peak, which accommodated 51.1% synthetic products (black plot) and only slightly decreased upon incubation with *E. coli* cells (green plot). However, the synthetic RNA set contained 33.4% of longer oligos up to the expected length (44 nt), which included 6 nt added to the tRF-pseudo sequence found inside *E. coli* cells (Figure 2d and Figure 4g). Endonuclease cleavage in the duplex formed with the participation of the added hexanucleotide led to the accumulation of longer RNAs than in the main pool (peak 2). Another endonucleolysis site (peak 3) was located in the second structural domain (Figure 6c), where the split resulted in a parallel accumulation of reads ending in the adjacent position (orange plot).

Culture-medium-mediated changes in the profile of tRF-Val_Rr_ (Figure 6e) also demonstrated the accumulation of fragments longer than the main pool (peaks 1 and 2): The stability of the 5′-ends belonging to the RNAs forming peak 3 and the presence of the second cleavage site increasing the number of fragments in peak 4. However, there are two features that were not observed for tRF-Lys and tRF-pseudo. First, in both sets of experimental data, there is a several-times-higher percentage of reads with new 3′-ends (peaks 6 and 7). Since both peaks correspond to the stem of the anticodon arm (Figure 6f) and are composed of thousands of identical reads, it is likely that they mainly appear due to endonuclease, rather than exonuclease, cutting. An alignment of reads from peak 6 indicates that 57.9 ± 7.4% of them have 5′-ends of peak 1. Reads from peak 7 are more diverse but contain the same 5′-ends in 28.1 ± 1.4% of the sequences, and 23.5 ± 1.3% of the oligonucleotides with 5′-ends correspond to peak 2. The second feature is the absence of 12- and 13-mers with 5′-ends of peak 5. This is due to their shortening from the 3′-ends to at least 10-mers, which were not taken into account in this work. Thus, exonuclease trimming of tRF-Val_Rr_, as identified by a decrease in the number of reads with untruncated 3′-ends, was confirmed by the “disappearance” of thousands (about 5%) of short reads with identical 5′-ends.

Although the set of tRF-Val_Pc_ synthetic products was highly heterogeneous (Figure 6h, black plot), it contained many full-length reads, which allowed us to confirm the accumulation of oligonucleotides with standard 5′- and 3′-ends (peaks 1 and 2 in Figure 6h, respectively). It is noteworthy that the nucleases attacked almost the same sites as in the secretomes of mixed populations (Figure 4a and Figure 6g), although the tRF_Pc_ folding was significantly different from that of tRNA-Val, at least at the site of 5′-end processing. In tRNA, cutting occurs in an optional loop, which, in tRF, base-pairs with a part of the acceptor helix, but still the adjacent phosphodiester bond was cut. Cleavage of the 3′-end took place at the junction of the Ψ-arm with a new duplex. The fragments found in the culture medium were only two nucleotides shorter with a cut in the acceptor stem. Such correspondence was also identified for the tRF-Lys 3′-end trimming (Figure 4c and Figure 6b) and for the processing of the 5′-ends of tRF-pseudo reads forming peak 2 (Figure 4g and Figure 6c). This similarity is the most evident for tRF-Val_Rr_, where endonuclease cleavage of oligos forming peaks 6 and 7 (Figure 6f) was witnessed by the presence of oligonucleotides with the same 5′- and 3′-ends in the growth milieu (Figure 4f).

Thus, although due to the premature termination of chemical synthesis we failed to trace a nearly pervasive 5′-end shortening of tRF-Val_Rr_ (Figure 4e), all other extensions turned out to be informative, and all the detected rearrangements were surprisingly reproducible. As a result, in contrast to the expected random digestion of synthetic RNAs by ubiquitous exonucleases, we observed their nonrandom cleavage by endonucleases in the culture medium with a relatively small truncation of the 3′-ends.

### 2.6. Alien tRFs Underwent Additional Structural Changes upon Entry into E. coli Cells but Partially Retain the Pattern of Extracellular Nuclease Cleavage

Fragments of all model oligonucleotides were found in *E. coli* cells in both experiments (Eco_in_oligos_1 and 2 in Supplementary Table S1), and their penetration efficiency was estimated as the percentage of imported tRFs of each type in the total number of such reads found inside and outside of the cells. The average values turned out to be as follows: 0.066 ± 0.033%, 0.072 ± 0.001%, 0.093 ± 0.038% and 0.277 ± 0.082% for tRF-Val_Rr_, tRF-pseudo, tRF-Lys and tRF-Val_Pc_, respectively. Therefore, only a minor fraction of extracellular RNAs can participate in regulatory events, if any. To compare the types of fragmentation of imported RNAs with those registered in the culture medium, we combined intracellular sets obtained in two experiments (N_c_ in Figure 6). The alignment of imported tRFs showed that the distribution profile of their 3′-ends roughly corresponds to that of synthetic analogs from the culture medium: The 3′-ends of tRF-Lys, tRF-pseudo and tRF-Val_Pc_ coincided with synthetic oligos by 90 – 99%, while the tRF-Val_Rr_ set had 30% truncated fragments of peak 6 in Figure 6e. Although the number of imported tRFs was very low, we profiled the 5′-ends for three of them with N_c_ > 50 to check for the presence or absence of further processing (red dotted plots in Figure 6). Exhibiting partial preservation of the extracellular cleavage pattern, all sets demonstrated additional changes: fragments from dominant peaks in extracellular samples (green plots of Figure 6) usually decreased in number, while the percentage of shorter fragments increased. In the set of tRF-pseudo, even a new peak (4) was formed due to the nuclease cleavage of longer synthetic oligos almost at the same site that was attacked in *P. copri* secreted tRNAs in mixed populations (Figure 6c, Figure 6d, Figure 4g and Figure 4h, respectively). Thus, it is likely that secreted RNAs undergo nonrandom cleavage by nucleases, which can be medium- and cell-specific.

### 2.7. Permutation of Synthetic Oligonucleotides Indicates Self-Ligation and Nuclease Cleavage of the Intermediate Circular Isoform in the Culture Medium

**Figure 7:**
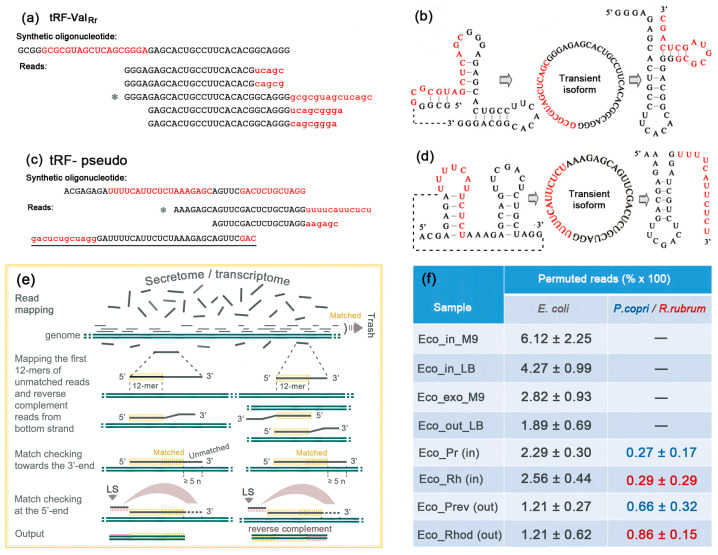
Synthetic analogs of tRFs underwent intramolecular permutations witnessing the existence of intermediate circular isoforms. Sequences with permutations in synthetic oligonucleotides are marked in red (**a**,**c**). Asterisks indicate the reads used for structural modeling (**b**,**d**). (**e**) Strategy used for bioinformatics search of permuted reads in RNA-seq data. (**f**) Average percentage (×100) of permuted reads estimated in quality-filtered datasets listed in Supplementary Table S1.

Alignment of the reads obtained upon the sequencing of synthetic analogs revealed several types of other modifications, including 5′- or 3′-flanking chimeras. Some of them have sequences derived from the *E. coli* genome and are definitely ligation products. This can happen either in the cells/medium or upon library construction stage. However, for the longest model oligonucleotides (tRF-Val_Rr_ and tRF-pseudo), intramolecular rearrangements were found. Since their appearance requires not only ligation but also cutting at another site (Figure 7a–d), they can be considered traces of intermediate cyclization—a process that, to our knowledge, has not yet been characterized in bacteria. Therefore, we developed an algorithm to search for permutations in RNA-seq datasets in order to estimate the frequency of their occurrence in natural transcriptomes and secretomes.

The two-step program (Figure 7e) operates with the genome of interest and a set of reads with a length ≤ 1000 nt. In the first step, reads that exactly match the genome are removed, and a file with unmatched reads is generated. In the second step, all reads from the new file are sequentially analyzed by scanning the genome in search of an exact match for the first 12-mers at the 5′-end. If detected on the upper strand, the program aligns the 3′-end of the read with the adjacent genome sequence and checks for the presence of an unaligned segment of the read upstream of the 5′-end in the genome. If the read matches the bottom strand, a reverse complement copy is obtained, and its 3′-end is aligned with the top strand. Reads with unmatching segments shorter than the specified size and reads with any deviations from the genomic sequence besides linear permutation were ignored (such as the underlined read in Figure 7c with GAC on both ends). As a result, when scanning the genomic sequence, rearranged reads mapped to the top strand have a permutation directed upstream of the ligation site (LS), while reads from the bottom strand are permuted in the opposite direction. Scanning of both strands of the genome is required to collect all permuted reads.

Twenty sets of quality-filtered RNA-seq data were implemented to evaluate the frequency of permutations and its dependence from bacterial growth conditions. In eight transcriptomes (Eco_in_M9_1/2, Eco_in_LB_1/2, Eco_Pr_1/2 and Eco_Rh_1/2; Supplementary Table S1), a total of 756 oligonucleotides ranging in size from 17 to 50 nt and the longest permuted segment of 36 nt were identified. Although most of them (532) were found in only one copy, the maximum number of identical oligonucleotides found in one experiment was 25. The set of permuted reads contains oligos derived from genes and intergenic spacers, but the main contribution was from 199 5S rRNA fragments (26.3%), which is significantly higher than the percentage of their correct isoforms in the datasets (4.00 ± 0.93%). Fragments of 16S- and 23S-rRNA were also slightly overrepresented (5.2% versus expected 2.98 ± 0.66%). The contribution of 132 permuted reads (17.5%) derived from tRNAs, on the contrary, was lower than expected (28.04 #xB1; 4.80%), while the presence of 59 (7.8%) permuted reads of sRNAs was almost at the expected level (6.80 ± 1.18%). Supplementary Table S2 demonstrates the genomic loci that produced permuted RNAs found in at least three of the four types of experiments. Among them, there are surprisingly many examples of an exact coincidence of LSs identified in completely different experiments. 

In twelve secretomes (Eco_exo_M9_1/2, Eco_out_LB_1-3, Eco_Prev_1-4 and Eco_Rhod_1-3 experiments; Supplementary Table S1), we found only 518 rearranged RNAs, and many of them originated from exactly the same genomic loci as intracellular oligonucleotides (Supplementary Table S3). Their size varied in nearly identical range as in the cellular sets (17–48 nt). On average, they were slightly shorter than intracellular rearranged fragments (28.7 and 33.1 nt, respectively), and the longest permuted segment was 32 nt in length. However, there is a big difference in composition from the intracellular set. Thus, the largest contribution (88 reads) was from 16S and 23S rRNAs (17.0%), which is close to the abundance of their non-permuted fragments in the culture medium (23.9 ± 7.76%). The same is true for sRNA fragments, the presence of which in non-permuted isoforms in the *E. coli* medium was very low (0.99 ± 0.04%), and only two rearranged reads were found. The amount of permuted tRNA fragments registered in the culture medium (5.2%) turned out to be much less than expected, based on the almost equal presence of their normal isoforms in the cells and medium (31.27 ± 6.03%), and rearranged 5S rRNAs fragments were represented by only three reads in the sets, where 2.78 ± 0.47% of their non-permuted isoforms were found. Thus, it is likely that the isomerization and/or secretion of tRFs and 5S rRNA fragments is biologically controlled.

At the last stage, we compared the occurrence frequency for permuted oligonucleotides in 8 datasets with cellular RNAs and 12 read libraries with secretomes obtained in different types of experiments (Figure 7f and Supplementary Table S1). The percentages of permuted oligos were calculated based on the number of unmapped reads. It is likely that they were higher within individually grown cells than in the secretomes (Figure 7f; Eco_in_M9/LB and Eco_out_M9/LB), although the difference is not statistically significant with Bonferroni correction [61] for multiple comparisons (LB and M9 media; *p* = 0.040). The percentage of permuted RNAs in the secretomes of the mixed populations was approximately in the same range as in pure *E. coli* culture, if the permuted RNAs produced by *P. copri/R. rubrum* are summed up with rearranged oligos of *E. coli* (Figure 7f; datasets Eco_out_LB, Eco_Prev (out) and Eco_Rhod (out)). Since the size of the *E. coli* RNA secretome estimated using the same datasets (Figure 3a) was significantly smaller than that of *P. copri*, it was interesting to find the larger percentage of permuted reads produced by *E. coli* (samples Eco_out_LB and Eco_Prev (out); Figure 7f). Most surprising is the about 1.5–2-fold decrease in the percentage of *E. coli* intracellular permuted RNAs in the presence of cohabiting bacteria, which only slightly contributed to their cellular abundance (datasets Eco_Pr and Eco_Rh versus Eco_in_LB). Thus, it can be speculated that the regulatory networks that support interspecies communications in bacterial consortia control the ring isomerization of some RNAs or, conversely, their elimination from cells by endonuclease cleavage.

## 3. Discussion

It is already well acknowledged that bacterial RNA fragments are in high numbers present in different environments, including human blood and other biological liquids [62]. However, their fate there, even in model conditions, has not been systematically evaluated so far, and to the best of our knowledge, this is the first study addressing the primary important questions in this area: what kind of alien RNAs are routinely taken up by *E. coli*, and how they are modified in culturing medium or recipient cells? Using the Zinder and Lederberg approach [63], which was suggested more than 70 years ago, we found RNA fragments belonging to *P. copri* or *R. rubrum* inside *E. coli* cells, and it was not surprising that most of them originated from tRNAs. Transfer RNA derivatives are widely known as regulatory molecules, which are currently the focus of research due to the importance and variety of their functions [28]. Thus, four tRFs imported by *E. coli* and affecting its growth dynamics were selected for sequence analysis at the stages of their persistence, both outside and inside of recipient cells. None of them belonged to the categories of ETS, ITS or tRNA halves, which have already been scrutinized in terms of functions [36,37,38,39].

Since tRNAs form stable secondary structures, they are naturally targeted by endonucleases for maturation. Thus, it was expected to find products of their endonucleolytic attack. Kumar and colleges [32] already documented the nonrandomness of their intracellular cleavage. In this work, we followed this process by using datasets that we previously obtained from mixed bacterial populations [47] and new RNA-seq data acquired with synthetic analogs of model tRFs. In both cases, we observed nonrandom fragmentation of extracellular oligonucleotides, thus indicating their preferential processing by endonucleases rather than exonucleases and, therefore, the presence of endonucleases outside bacterial cells. Since the synthetic analogs lacked modifications typical for natural tRNAs and, in some of them, the most stable structures differed from those formed in the “cloverleaf”, it was surprising that certain endonuclease attack sites of the model tRFs coincided with those identified in the natural secretomes (Figure 4 and Figure 6). It is well known that tRNA precursors are extensively processed by Rnase P and RNAse Z, which remove excessive nucleotides from the 5′- and 3′-ends, respectively [64], while single-strand-specific RNAse E can be involved in the cleavage of polycistronic tRNA transcripts [65]. All of these processes are part of tRNA maturation, as such. They were detected for tRF-Val_Rr_, tRF-Val_Pc_ and tRF-Lys in the secretomes of mixes populations (Figure 4) and/or in the sets obtained from *E. coli* growth media cultured in the presence of synthetic oligos (Figure 6). The previously described Dicer-like endonuclease cleavage in the Ψ-arm loop [36] is part of the tRF maturation process. It was found for both tRFs originating from the 3′-ends of tRNAs (tRF-Lys and tRF-Val_Pc_) having the same sequence in the Ψ-loop (peaks 3 in Figure 6a,b,g,h). However, we also observed intramolecular cleavage sites located in the stems of all model tRFs (Figure 6), thus indicating a more extensive processing of functional tRFs by yet unknown endonucleases.

The biological significance of the endonucleases involved in the cleavage of circular RNAs (circRNAs) with the generation of rearranged (permuted) oligonucleotides is not clear so far. In eukaryotes, covalently closed molecules represent a recently discovered class of endogenous oligonucleotides, which are formed through a back-splicing mechanism from linear transcripts [66,67,68]. Numerous circRNAs have been identified by means of high-throughput sequencing techniques in combination with computational analysis [69], i.e., the same way as performed in this study. Many particular examples have been intensely studied in terms of their functions [70,71]. Their known biological roles include acting as microRNAs [72] or protein “sponges” [73,74]. They are involved in the regulation of immune responses to various pathogens [75] and possess high diagnostic value as biomarkers of cancer [76]. Due to their high stability, specially designed circRNAs are used as specific biotechnological tools [77,78]. However, little is known about the natural occurrence of circRNAs in bacteria. Thus, Roth et al. [79] identified 290 examples of circularly permuted group II introns (ribozymes) in the genomes/transcriptomes of several bacterial taxa, indicating the universality of back-splicing for circular isoform biogenesis. However, splicing, as such, is a relatively rare event in bacteria. Thus, using a bioinformatics search for permuted oligonucleotides in 23 bacterial transcriptomes of *E. coli* and *Enterococcus faecalis*, Innocenti et al. [80] identified 2660 genomic clusters producing permuted RNAs with a length from 44 to 97,246 nt (reads and contigs). Although we searched only for permutation events in short reads (17–50 nt) with no sequence deviations other than linear rearrangements, the published set [80] contains 535 permuted reads identified in our study. Of these, 281 overlap with regions shorter than 175 bp, which may represent individual reads, and 213 (75.8%) of them correspond to 5S rRNA sequences. Thus, it is likely that 5S rRNA fragments specifically undergo circular isomerization by yet unknown ligases, which have surprisingly high specificity, since many of their action sites, exactly identified by the left border (LS in Figure 7e) of the permuted segment, completely coincided in different experiments (Supplementary Tables S2 and S3).

Starting this study, we already knew that all extracellularly added oligonucleotides penetrate into bacteria [47]. Two approaches in this work made it possible to assess the efficiency of penetration quantitatively. The co-cultivation of bacteria in separate compartments of the common chamber provided a chance to quantify the percentage of *P. copri* and *R. rubrum* oligos imported by *E. coli* cells (Figure 2b,c). Subtracting the background caused by the ectopic presence and reads matching to both genomes in *E. coli* monocultures resulted in values of 0.085 ± 0.023% and 1.115 ± 0.213% for *P. copri* and *R. rubrum*, respectively. They vary greatly, and in the case of *R. rubrum*, the percentage may be overestimated, as reads classified as “commons” significantly contributed to the “imported” RNAs (Figure 2c). In each experiment with synthetic oligos, their amount was quantified both inside the cells (Eco_in_oligos_1/2) and in the culture medium (Eco_out_oligos_1/2). Then, the percentage of imported molecules from their total number in the sample was calculated. For tRF-Lys and tRF-pseudo, they correspond to the value that was calculated above for RNAs secreted by *P. copri* (0.093 ± 0.038% and 0.072 ± 0.001%, respectively); the percentage of imported tRF-Val_Rr_ was lower (0.066 ± 0.033%), but for tRF-Val_Pc_, it reached one-third of the value calculated for *R. rubrum* secretome (0.277 ± 0.082%). This means that the ability of *E. coli* to import RNA depends on the features of particular oligonucleotides, and a reasonable range can be roughly estimated between 0.03% and 1.3%.

Synthetic oligos added at a known concentration (1 μM) to the bacterial growth medium can also be considered calibration spikes, which made it possible to roughly assess the concentration of extracellular RNAs. This was performed using the total number of QC reads obtained in the Eco_out_oligos_1/2 experiments (Supplementary Table S1) and the portion of the reads mapped to the four tRFs in them (indicated in Figure 6). As a result, the average concentration of extracellular RNAs during the stationary growth of *E. coli* in M9 medium turned out to be 0.69 ± 0.02 μM. Thus, the concentrations of exogenous RNAs in the range of 0.5–2.0 µM, which caused different cell responses at the logarithmic and stationary phases of growth, are apparently physiologically relevant for studying the mechanisms of their signaling action. Since *R. rubrum* has approximately the same size of RNA secretome as *E. coli* (Figure 3b), the stimulatory effect observed in growth experiments performed with media obtained from *R. rubrum* culture (Figure 1e–h) was caused by approximately 0.14 μM and 0.28 μM concentrations of exogenous RNAs. In the case of *P. copri*, with a larger RNA secretome (Figure 3a), the concentration of potential signaling RNAs in conditioned media was higher, and this may be the reason why the effect was more pronounced. It is important, however, that, in both cases, we observed stimulatory, rather than inhibitory, effects, which were expected based on the data obtained in the ENCODE project [61], as well as in our previous study using parallel growth of the same bacteria in either monocultures or mixed populations [48]. As a result, an expectedly slower increase in the optical density was registered in the mixed populations of *E. coli* + *P. copri* and *E. coli* + *R. rubrum* compared to the sum of densities in individually grown populations, while in the symbiotic pair *P. copri* + *R. rubrum*, an opposite effect was observed.

The two methods applied differed in the type of influence on the culture of *E. coli*: introduction of only metabolites of other bacteria or of their living cells. This is reminiscent of experiments carried out with bacteria that are in a predator–prey relationship (*Myxococcus xanthus*–*E. coli*), in which the predator transcriptome reacted much stronger to the presence of killed than alive *E. coli* cells [55,56]. We previously found out that in the presence of *P. copri* or *R. rubrum*, *E. coli* alters the profile of secreted RNAs [47]. Now, we observed that even the size of *E. coli* RNA secretome was reduced upon cultivation with *P. copri* or *R. rubrum* (Figure 3a,b). Intuitively, this also indicates successive competition of these two bacteria against *E. coli*. Therefore, the question arises, how can the stimulating effect of their metabolites on the growth of *E. coli* be explained, given that the influence of its own metabolites, which could contribute to its adaptation to a nutrient-depleted broth, did not provide such a benefit (Figure 1a,b)? Illustrating a biological phenomenon that is difficult to explain, this suggests a closer-than-expected, and perhaps even direct, interaction between bacterial secretomes. The presence of a stimulating effect in growth experiments with the addition of exogenous tRFs (Figure 5) indicates the participation of RNAs in this process but does not necessarily require their penetration into recipient cells.

All in all, our work indicates the importance of studying all stages of RNA-mediated signaling in bacterial consortia. The exceptional plasticity of RNAs, provided by different mechanisms of their processing and isomerization, underlies the huge potential for the impact of secreted RNA on the metabolism and viability of symbionts in ecosystems. Further study of their signaling and bacteriostatic functions requires the development of new methods of experimental and bioinformatics analysis to search for endonucleases capable of nonrandom cutting of RNAs in secretomes and enzymes necessary for the relaxation of their cyclic isomers. New ideas are needed to estimate the role of such isomers in bacterial transcriptomes and secretomes.

## 4. Materials and Methods

### 4.1. Bacterial Strains and Growth Conditions

**Figure 8:**
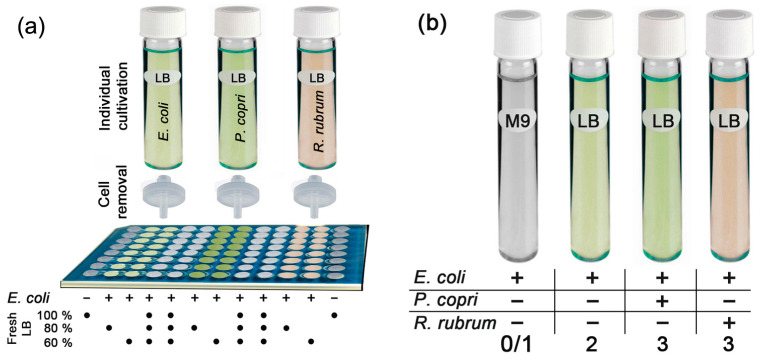
(**a**) Strategy of the experiment with cultivation in conditioned LB media. The “−” indicates plate wells containing fresh LB medium without inoculation, and “+” shows wells inoculated with *E. coli*. (**b**) Biological samples of *E. coli* cultures, grown alone or in combination with *P. copri* or *R. rubrum*, which were used for RNA-seq analysis.

The laboratory strain of *E. coli* K12 MG1655, together with *P. copri* and *R. rubrum* ATCC 11170 strains obtained from the DSMZ collection (DSM 18205 and DSM 467 at https://www.dsmz.de/collection/catalogue/microorganisms (accessed on 7 July 2023), was used in the study. *E. coli* strain K12 MG1655 was taken as a taxon, with the deepest understanding of the coding potential of its genome and the composition of the RNA secretome. The choice of *P. copri* was due to the dominant presence of bacteria of this genus in the second enterotype microbiome of the human intestine [59], and *R. rubrum* ATCC 11170 was selected as a species with presumably the same type of interspecific relationship with *E. coli* as in *P. copri*. For experiments with *P. copri* and *R. rubrum*, culturing was conducted in Luria-Bertani medium (10 g/L of tryptone, 10 g/L of NaCl, 5 g/L of yeast extract and 0.4 g/L of L-cystein-HCl, pH 7.0) in anaerobic conditions (1–1.5% oxygen). To ensure a low level of oxygen, the culture medium (pH 7.0) was heated in a water bath (10–15 min) and cooled under CO_2_ flow. Hungate tubes (Figure 8a,b) and chamber compartments (Figure 2a) were purged with N_2_, filled with culture medium and tightly closed by screw caps with rubber gaskets.

The strategy used for culturing bacteria in conditioning media when assessing the interspecies relationships of *E. coli* with *P. copri* and *R. rubrum* is shown in Figure 8a. Samples prepared as illustrated in Figure 8b were used to evaluate the relative amount of RNA secreted by different bacteria, to assess the presence of selected tRFs in *P. copri* and *R. rubrum* secretomes and to determine the type of their fragmentation in the culture medium.

Filled culturing vessels were sterilized in an autoclave for 20 min at 121 °C. Individual cultivation of *E. coli* was carried out either under aerobic or anaerobic conditions to obtain control samples for different types of experiments described below. M9 mineral medium (3 g/L of KH_2_PO_4_, 6 g/L of Na_2_HPO_4_, 0.5 g/L of NaCl, 1 g/L of NH_4_Cl, 2 mM of MgSO_4_, 0.1 mM of CaCl_2_ and 0.5% glucose) was used where possible to avoid residual contamination of samples with LB-born RNAs. Fresh M9 or LB medium was inoculated with 1:1000 or 1:4000 *E. coli* cultures overnight, respectively, and incubated at 37 °C for 12 h (M9) or 8.5 h (LB) prior to RNA extraction. Growth conditions for all experiments are indicated in Supplementary Table S1. 

### 4.2. Cultivation of E. coli in Conditioned LB Media

The ability of metabolites secreted by *P. copri* and *R. rubrum* to affect the growth of *E. coli* was checked through the addition of cell-deprived LB medium after culturing of *E. coli*, *P. copri* and *R. rubrum*. Bacteria of all the three types were anaerobically grown overnight in Hungate tubes in Luria-Bertani medium (LB), containing tryptone (10 g/L), NaCl (10 g/L), yeast extract (5 g/L) and L-cystein-HCl (0.4 g/L), pH 7.0. To obtain comparable growth dynamics of all the three strains, 10 mL of fresh LB was inoculated with a previously optimized [47] ratio of overnight cultures: 1:10 for *P. copri* or *R. rubrum* and 1:1000 for *E. coli*. Followed by 24 h of incubation at 37 °C, with gentle agitation, bacterial cells were pelleted by 15 min of centrifugation (3000 rpm, +4 °C). Supernatants of conditioned media were collected and filtered through a 0.22 µm syringe filter (Figure 8a). Overnight-grown *E. coli* bacteria were inoculated 1:100 in freshly prepared LB with the addition of 20 or 40% of conditioned media and cultured in 0.2 mL wells of plastic 96-well plates (TC Plate Well, Suspension, F; Sarstedt AG & Co, Nümbrecht, Germany) in Synergy H1 Hybrid Multi-Mode Reader (BioTek Instruments Inc, Berlin, Germany), at 37 °C, with agitation. The optical density of *E. coli* culture was monitored (λ = 650 nm) every 30 min for at least 30 h.

### 4.3. Co-Culturing of Bacteria in Membrane-Separated Compartments

*E. coli* was grown with *P. copri* or *R. rubrum* in LB medium supplemented with 0.4 g/L of L-cysteine-HCl in chamber compartments (Figure 2a) that were separated by an impermeable for bacteria membrane (Millipore Express PLUS Membrane Filter; pore diameter 0.22 µm) (Merck, Readington, NJ, USA). This allowed to maintain equal growth conditions for co-culturing bacteria and ensured interexchange of metabolites and macromolecules between cultures. Since Millipore membranes have different structures of the two surfaces, two layers of membranes with smooth surfaces turned inward were used to ensure the same conditions for the diffusion of solutes from both compartments. The inability of the bacteria to cross the membrane was confirmed using a combination of inoculated and non-inoculated *E. coli* compartments, followed by the plating of liquid samples on LB agar Petri dishes. Membrane permeability for nucleic acids was confirmed by PCR, using the *E. coli* specific *appY* gene as a target for amplification. LB-filled compartments of the chamber were inoculated with *P. copri* cultures (1:10) grown for 48 h at 37 °C or *R. rubrum* cultures (1:10) grown at 30 °C under LED lamp illumination. The *E. coli* inoculum was grown for 24 h and added to the chamber in a 1:4000 ratio. LED lamp illumination was used in all experiments with *R. rubrum*. Following incubation, short RNAs were isolated from the bacterial cells and supernatants.

### 4.4. Effect of Exogenous Oligonucleotides on E. coli Growth

Four RNA probes corresponding to tRNA gene fragments encoded in the genomes of *P. copri* and *R. rubrum* were synthesized in triplicate and PAGE purified from substrates and components of chemical synthesis by Syntol LLC (Moscow, Russia). They were obtained in the amount ranging from 2 to 16 optical units and were not subjected to fractionation to account for possible effects from full-sized and shortened variance. Purified synthetic products were dissolved in water to a stock concentration of 100 pmol/µL and stored in a freezer until use. The experiments were carried out in at least three biological trials with different inocula, using at least two portions of synthetic oligonucleotides. Overnight *E. coli* culture was diluted 1:1000 and seeded in 96-well plates (200 µL), followed by the addition of synthetic model RNAs at a concentration range of 0.5–5 µM. Bacteria were cultured for 24 h in M9 medium supplemented with 0.5% glucose in the presence of 5% oxygen, with agitation. The same inoculum without oligos was used to prepare control samples. The optical density was monitored at 650 nm in an automated mode every 20 min. Growth curves were plotted in SigmaPlot version 11.0. The areas under the dynamic curves in different phases of cultivation were assessed with GraphPad Prizm 5 software [60].

### 4.5. Evaluating the Effects Posed by E. coli on the Integrity of Exogenous Oligonucleotides

To evaluate the stability of exogenous oligonucleotides in the culturing milieu of growing *E. coli*, 5 mL of M9 medium + 0.5% glucose was inoculated with 1:1000 overnight *E. coli* culture, with the addition of one type of synthetic RNAs per tube to a final concentration of 1 pmol/µL. After 12-h anaerobic culturing in Hungate tubes, the cultures were combined; 3 mL was consequently passed through two 0.22 µm syringe filters, and 2.5 mL was used for the isolation of extracellular sRNAs. Cells from the remaining 17 mL mixture were pelleted via centrifugation (3000 rpm, +4 °C), washed twice with PBS (8.0 g/L of NaCl, 0.2 g/L of KCl, 1.44 g/L of Na_2_HPO_4_ and 0.24 g/L of KH_2_PO_4_, pH 7.4) and used for the isolation of cellular RNAs.

### 4.6. RNA Isolation

Fractions of short RNAs were isolated from bacterial cells and culturing media, using basically the same protocols as described previously [46,47]. In brief, the cellular sRNA fraction was obtained from bacteria harvested by centrifugation (3000 rpm, +4 °C) from 5 to 20 mL, and the pellet was washed with sterile PBS. If cells were obtained from the 5 mL culture, they were treated with 1 mL of TRIzol reagent (Thermo Fisher Scientific, Waltham, MA, USA). They were thrice frozen in liquid nitrogen and thawed at 37 °C to favor cell disruption. Then, 200 µL of chloroform was added, and the sample was incubated for 10 min at room temperature and centrifugated for 15 min at 12,000 rpm (+4 °C). The sample was enriched with short RNAs, using a mirVana miRNA Isolation Kit (Thermo Fisher Scientific, USA). For the isolation of extracellular RNAs, 2.5 mL of supernatant obtained after centrifugation of bacterial culture at 3000 rpm (+4 °C) was sequentially passed through two 0.22 µm syringe filters and treated with a Qiagen miRNeasy Serum/Plasma kit (Qiagen, Venlo, The Netherlands) according to the manufacturer’s protocol. The concentration of RNA samples was determined on a Nanopore ND-1000 spectrophotometer and Qubit 3 fluorometer (Thermo Fisher Scientific, USA). 

### 4.7. Preparation of Sequencing Libraries and Sequencing

Libraries for sequencing on the Torrent PGM platform were prepared using the Ion Total RNA-Seq Kit v2 (Thermo Fisher Scientific, USA) according to the manufacturer’s protocol, but with modifications. Following adapter ligation, reverse transcription and amplification, the samples were purified using a Monarch PCR DNA Cleanup Kit (New England Biolabs, Ipswich, MA, USA) and run in 6% PAAG. The gels were stained with EtBr (1 µg/mL), and the area of interest on the gel, spanning from ~90 to 123 bp, was cut out, minced, soaked in elution buffer from NEBNext Multiplex Small RNA Library Prep Set for Illumina (Set 1) (New England Biolabs, USA), incubated overnight, precipitated with ethanol, washed and dissolved in sterile deionized water. The libraries were normalized to a concentration of 100 pM and sequenced on Ion Torrent PGM (Thermo Fisher Scientific, USA). The library of synthetic fragments was prepared from combined equimolar amounts of RNA probes (1 µL of 100 µM solution each). All subsequent manipulations were as described above for experimental samples.

### 4.8. Data Processing

The sets of reads obtained from sequencing were processed on the Galaxy platform, server usegalaxy.org (accessed on 7 July 2023) [80]. BAM files were converted to FASTQ, using the Convert, Merge, Randomize tool, and then filtered using Filter by Quality tool (quality cutoff = 20; percent of bases of this quality in each read = 90) and converted to FASTA format. Transcriptomes obtained from cells grown in LB were filtered against sequence reads belonging to the medium, using the previously obtained dataset LB_medium_1/2 (Supplementary Table S1), as was described in [47]. Although the adapter sequences were removed by the Ion Torrent Suite Software v.5 during base calling, the obtained reads were additionally checked in Excel for their residual presence and removed if at least 13 nucleotides of their terminal sequences were found (absent in the genomes of all model bacteria). 

### 4.9. Bioinformatics Analysis

All 27 RNA-seq datasets, of which 11 were obtained in this study (PRJNA687658), 12 were used previously [47] and 4 were received for another project GEO GSE221667, are available from NCBI GEO and NCBI SRA, as indicated in Supplementary Table S1.

#### 4.9.1. Search for RNAs Secreted by *P. copri* or *R. rubrum* and Imported by *E. coli*

Four datasets (Eco_Pr_1/2 and Eco_Rh_1/2 in Supplementary Table S1) with intracellular RNAs isolated from *E. coli* cells grown in an adjacent compartment with *R. rubrum* or *P. copri* were used to find alien RNA fragments imported by *E. coli*. Following the filtration from LB-borne sequences and residual presence of adaptors, 16–50 nt long reads were mapped to the genomes of *E. coli* and co-cultured bacteria, using the PrMis3MatcherOutDiffLen program (https://mathcell.ru/prog/ReadTools.rar (accessed on 7 July 2023)), permitting it to collect reads that match the target genome with a specified number of mismatches (1–3). To estimate the percentages of reads produced from different bacteria, the RNA-seq datasets were sorted into three categories: Those that exactly matched the genomes of *E. coli* or the cohabiting bacterium and those that exactly matched both genomes. Their percentage was estimated per the number of 16–50 nt long reads in each experiment separately and averaged. To collect potentially imported RNAs, we removed reads with 0 and 1 mismatches to the genome of *E. coli* and collected reads matching only the genomes of *P. copri* or *R. rubrum*. To estimate the false-discovery rate, four datasets obtained from individually grown *E. coli* in M9 (Eco_in_M9_1/2) or LB (Eco_in_LB_1/2) medium were evaluated in the same way. To select candidates for experimental evaluation, all 198 RNA fragments with a high probability of being secreted by *P. copri* and 28 oligonucleotides that may be of *R. rubrum* origin were aligned with the genome of *E. coli*, using NCBI Microbial Nucleotide BLAST, “somewhat similar sequences” mode and default options. Four tRNA fragments were chosen due to their belonging to tRFs and low level of matching to the genomic sequence of *E. coli*.

#### 4.9.2. Evaluation of the Relative Sizes of *E. coli*, *P. copri* and *R. rubrum* Secretomes in Mixed Populations

Seven previously published datasets (Eco_Prev_1-4 and Eco_Rhod_1-3, Supplementary Table S1) [47] were used to assess the efficiency of RNA secretion by three model bacteria. To improve the efficiency of the secreted RNA matching, length-sorted oligonucleotides 16–50 nt long were trimmed in Excel by 1 nucleotide from the 5′-end and 3 nucleotides from the 3′-end. Thus, the compared sets contained 12–46 nt long sequences. Then, the PrMis3MatcherOutDiffLen program was used to estimate the percentages of reads produced from different bacteria, as described above. They were calculated per the total number of trimmed reads and averaged over 4 and 3 independent experiments. Five datasets obtained from individually grown *E. coli* in M9 (Eco_exo_M9_1/2) and LB (Eco_out_LB_1-3) medium were used as controls and evaluated in the same way to estimate the false-discovery rate. Since 4 experiments with bacteria grown in aerobic conditions in M9 medium were required for statistical assessment, while all other datasets were related to anaerobically cultured populations, to account for the possible effects of growth conditions on the *E. coli* transcriptome and secretome, 2 additional control datasets (Eco_in_LB_2 and Eco_out_LB_3) were obtained. Their analysis did not reveal any difference from samples isolated from anaerobically grown cultures; therefore, the data from experiments Eco_in_LB_2 and Eco_out_LB_3 were averaged with Eco_in_LB_1 and Eco_out_LB_1/2, respectively. 

#### 4.9.3. Assessing Fragmentation of Selected RNAs in the Medium of Mixed Populations

The same 7 datasets (Eco_Prev_1-4 and Eco_Rhod_1-3, Supplementary Table S1) [47] were used to find fragments of Lys and Val tRNAs of *R. rubrum*, as well as Val and pseudo-tRNAs of *P. copri* in natural secretomes of mixed bacterial populations. Extracellular RNAs, secreted by *E. coli* grown individually in either M9 or LB media (Eco_exo_M9_1/2 and Eco_out_LB_1-3), were used as controls (Supplementary Table S1). Four datasets obtained from mixed populations *E. coli*–*P. copri* and 3 sequence libraries from the *E. coli*–*R. rubrum* pair were pooled. Reads that mapped to the genome of *E. coli* with 0 and 1 mismatch were removed using the PrMis3MatcherOutDiffLen program, and unique 12-mers present in the context of the chosen tRNA fragments but absent in the *E. coli* genome were implemented as markers of corresponding tRNAs. Detected fragments were aligned using the Clustal Omega multiple sequence alignment tool on the EMBL-EBI portal. The positioning of their 5′- and 3′-ends was quantitatively determined using sorting and searching options of Microsoft Excel. Selected reads were sorted by length in 39 groups, from 12 to 50 nt, followed by the search in each group for reads exactly matching the sequences of target tRNAs and estimating the number of reads with particular 5′- and 3′-ends.

#### 4.9.4. Assessing Fragmentation of Synthetic Analogs of Model tRFs in the Cells and Medium of *E. coli* Monoculture

Five datasets (Synthetic oligos, Eco_in_oligos_1/2 and Eco_out_oligos_1/2, Supplementary Table S1) were used to evaluate the integrity of synthetic oligos added to the growing populations of *E. coli* cells. The control sample (synthetic oligos) reflects the distribution of the 5′- and 3′-ends in synthetic tRFs. The positions of these ends after cultivation were assessed for synthetic oligos both found in M9 medium (Eco_out_oligos_1/2) and those that entered *E. coli* cells (Eco_in_oligos_1/2). This was performed almost in the same way as described in Section 4.9.3, except that the search was directed to only 35–46 nt synthetic oligos that differed significantly from *E. coli* transcripts in terms of sequences (Figure 2d). This required neither the removal of *E. coli* RNAs nor an analysis of reads exceeding original sizes. The distribution of the 5′- and 3′-ends for all 4 tRFs was assessed in all 4 experiments independently, and their location with a resolution of 1 nt was averaged between replicates.

#### 4.9.5. Evaluating Circular Permutations of Synthetic Analogs and RNA Fragments of *E. coli*

Subroutine IdentifyCircularRna (https://mathcell.ru/prog/ReadTools.rar (accessed on 7 July 2023)) was implemented to identify circularly permuted synthetic analogs of tRFs in the sets Eco_out_oligos 1/2, as well as to evaluate the frequency of permutations in 20 other datasets (Supplementary Table S1). The strategy applied in the algorithm IdentifyCircularRna is illustrated in Figure 7e and explained in the main text. It detects rearrangements with a minimum transfer of 4 nt, but in this study, we analyzed permutations with relocated segments of at least 5 nt. After removing all the reads mapped to the bacterial genomes present in a particular experiment, the algorithm works with each read separately, scanning the genome for an exact match with the first 12-mer at the 5′-end. If marker 12-mers match to the top strand, the program aligns the 3′-end of the read with the adjacent genome sequence and checks for the presence of an unaligned segment of the read upstream of the 5′-end in the genome. This allows for the identification of the relocation from the 5′-end to the 3′-end. If the 5′-terminal 12-mer matches the bottom strand, then the algorithm reverse complements its sequence, aligns the new 3′-end with the adjacent genome sequence of the top strand and checks for the presence of an unaligned segment upstream of the new 5′-end. This allows for the identification of the relocation from the 3′-end to the 5′-end. Therefore, to detect permutations in both directions for 12-mers matching to the top and bottom strand, both strands of the genome were scanned, and reads containing any other deviations from the genomic sequence were ignored. At the output of each genomic strand scanning, the program provides sequences and the “left borders” (ligation sites, LS) of all genomic loci, which may produce permuted oligos. To assess their relative positioning, the coordinates obtained from scanning the bottom chain were converted to their positions on the top strand. This profiling provided a chance to compare the genomic distribution of the revealed genomic loci with data obtained by Innocenti et al. [80]. To assess the frequency of permutations, rearranged oligos were recovered from the output genomic sequences, and their multiplicity with copy number was determined in each experiment separately, avoiding multiple counting of identical reads mapped to paralogous genes.

### 4.10. Statistics

The statistical significance of values obtained from at least three biological experiments was assessed using the Student’s *t*-test in the SigmaPlot version 11 software package (http://www.sigmaplot.co.uk/products/sigmaplot/statistics.php (assessed on 7 July 2023)). The One-Sample *t*-test was used to calculate SEMs, which show the deviations in the experimental data presented in the plots. The Compare Two Groups option in the same package was implemented to estimate the statistical significance of differences if any. The Bonferroni correction, used to evaluate the significance of the difference in the percentages of permuted oligonucleotides inside and outside of *E. coli* cells, was estimated at https://www.easycalculation.com/statistics/bonferroni-correction-calculator.php (accessed on 7 July 2023).

## Data Availability

The datasets presented in this study are available in NCBI GEO Database (GSE221667) and NCBI SRA Database (BioProject PRJNA687658).

## Supplementary Materials

The following supporting information can be downloaded at https://www.mdpi.com/article/10.3390/ijms241612960/s1.
