# Supplementary material for: The Fate and Functionality of Alien tRNA Fragments in Culturing Medium and Cells of Escherichia coli
Source: Int J Mol Sci. 2023 Aug 19;24(16):12960. doi: 10.3390/ijms241612960 (PMC10455298; doi:10.3390/ijms241612960)
Supplement: Supplementary file 1 [file ijms-24-12960-s001.zip › Table_S1.pdf]

Table S1 Sequence reads sets used for analysis

Sample	Number of reads		Growth conditions	Description and availability
	Before QC	After QC		
Eco_Pr_1	2,629,571	1,563,678	LB medium, anaerobic growth	RNAs isolated from *E. coli* cells after their co-cultivation with *P. copri* or *R. rubrum* in membrane-separated compartments within common chamber. This study NCBI BioProject PRJNA687658
Eco_Pr_2	864,537	652,497		
Eco_Rh_1	1,301,404	1,028,350		
Eco_Rh_2	1,176,395	681,123		
Eco_Prev_1	1,532,963	1,290,466	LB medium, anaerobic growth	RNAs isolated from growth medium after *E. coli* combined cultivation in Hungate tubes with *P. copri* or *R. rubrum.* NCBI BioProject PRJNA687658 sample names: Eco_Prevot replicate 1, Eco_Prevot replicate 2, Eco_Prevot replicate 3_NaOH, Eco_Prevot replicate 4_NaOH, Eco_Rhod replicate 1, Eco_Rhod replicate 2, Eco_Rhod replicate 3_NaOH
Eco_Prev_2	937,975	662,605		
Eco_Prev_3	2,377,939	2,097,966		
Eco_Prev_4	711,408	447,987		
Eco_Rhod_1	1,625,146	1,429,628		
Eco_Rhod_2	807,732	644,415		
Eco_Rhod_3	2,123,797	998,024		
Eco_in_M9_1	1,546,157	996,435	M9 medium, aerobic growth	RNAs isolated from *E. coli* cells after their individual growth. NCBI GEO GSE221667
Eco_in_M9_2	2,494,971	1,528,878		
Eco_in_LB_1	1,320,485	299,167	LB medium, anaerobic growth	RNAs isolated from *E. coli* cells after their individual growth. NCBI BioProject PRJNA687658
Eco_in_LB_2	2,305,864	1,589,066	LB medium, aerobic growth	RNAs isolated from *E. coli* cells after their individual growth. This study NCBI BioProject PRJNA687658
Eco_out_LB_1	1,677,610	1,484,046	LB medium, anaerobic growth	RNAs isolated from growth medium of individually cultured *E. coli.* NCBI BioProject PRJNA687658 samples: Eco_out replicate 1 Eco_out replicate 2
Eco_out_LB_2	1,168,144	1,168,144		
Eco_out_LB_3	752,428	401,740	LB medium, aerobic growth	RNAs isolated from growth medium of individually cultured *E. coli* This study NCBI BioProject PRJNA687658

Eco_exo_M9_1	1,832,829	890,000	M9 medium, aerobic growth	RNAs isolated from growth medium of individually cultured *E. coli.* NCBI GEO GSE221667
Eco_exo_M9_2	1,220,822	737,402		
Synthetic oligos	700,677	609,191	-	Synthetic RNAs sequenced following chemical synthesis.<br>This study<br>NCBI BioProject PRJNA687658
Eco_in_oligos_1	1,415,935	984,031	M9 medium, anaerobic growth	RNAs isolated from *E. coli* cells after culturing in the presence of synthetic RNA oligonucleotides.<br>This study<br>NCBI BioProject PRJNA687658
Eco_in_oligos_2	2,010,978	1,502,859		
Eco_out_oligos_1	1,258,906	1,085,476	M9 medium, anaerobic growth	RNAs isolated from growth medium after culturing of *E. coli* in the presence of synthetic RNA oligonucleotides.<br>This study<br>NCBI BioProject PRJNA687658
Eco_out_oligos_2	1,726,464	1,261,818		
LB_medium_1	99,347	75,354	-	RNAs isolated from uninoculated LB medium.<br>NCBI BioProject PRJNA687658 sample names:<br>LB_medium,<br>LB_medium_NaOH
LB_medium_2	2,728,221	2,034,497		
