# Supplementary material for: The Fate and Functionality of Alien tRNA Fragments in Culturing Medium and Cells of Escherichia coli
Source: Int J Mol Sci. 2023 Aug 19;24(16):12960. doi: 10.3390/ijms241612960 (PMC10455298; doi:10.3390/ijms241612960)
Supplement: Supplementary file 1 [file ijms-24-12960-s001.zip › Table_S2.pdf]

Table S2 Genomic origins of permuted oligonucleotides found in cells from at least three of the four types of experiments

Indicated Genomic positions correspond to the ligation sites (LS) in the genomic loci as shown in **Figure 7e**.
Depending on the scanned strand and the localization of the LS found in the oligos, four types of rearrangements are possible:

1. Anchor 12-mer (upper case letters) was found upon **top** strand scanning and matched to the **top** strand (code ++).

Example: →

Sequence in the genome 228621 (+): 5′-cgttgagctaaccgGTACTAATGAACcgtgaggcttaacctt-3′
Sequence of read: 5′-GTACTAATGAACcgtgaggcttaaccttcgttgagctaaccg-3′

The green segment and arrow here and below indicate the permutation found in the read and its direction.

2. Anchor 12-mer was found upon **top** strand scanning and matched to the **bottom** strand (code + -).

Example:

Sequence in the top strand of the genome: 696457(+) 5′- ggaatgccggaatcaGAATCCGGTGCCttaccgcttggcgatac-3′
Permutation: 5′-GAATCCGGTGCCttaccgcttggcgatacggaatgccggaatca-3′
Read (reverse complement): 5′- tgattccggcattccgtatcgccaagcggtaaGGCACCGGATTC-3′ ←

3. Anchor 12-mer was found upon **bottom** strand scanning and matched to the **bottom** strand (code - -).

Example: →

Sequence in the bottom strand of the genome 696499(-): tatcgccaagcggtaaggcaccggattcTGATTCCGGCATtccg
Read: 5′- TGATTCCGGCATtccgtatcgccaagcggtaaggcaccggattc-3′

4. Anchor 12-mer was found upon **bottom** strand scanning and matched to the **top** strand (- +).

Example:

Sequence in the bottom strand of the genome 228833 (-): 5′-taccatcggcGCTACGGCGTTTcacttctgagttcggcatgg-3′.
Permutation: 5′-GCTACGGCGTTTcacttctgagttcggcatggtaccatcggc-3′
Read (reverse complement): 5′-gccgatggtaccatgccgaactcagaagtgAAACGCCGTAGC-3′ ←

● Genomic coordinates of LS coinciding (±10 bp) in different experiments are marked in the table with the same colors.
● LS positions found on opposite strands, which may be indicative of bidirectional permutations from the same ligation site, are marked in blue.
All permutation events found in at least 3 of the four types of experiments are listed, including those found in RNA fragments of homologous genes (tRNA, promoter regions of 16S rRNA and its coding sequence). However, the products of long 23S rRNA genes and 5S RNA fragments with a large number of permutation points are represented by the genes of only one operon (*rrlH* and *rrfH*, respectively).
● Coordinates of permuted fragments registered in multiple copies are shown in bold.

N	Type of genomic region	Ge-ne(s)	Strand	Gene borders		The genomic coordinate of the ligation site as detected in different types of experiments				Co-de	Examples of oligos, showing uni- and bidirectional permutation, as well as predominant location of ligation sites (marked red).
				Left	Right	Eco_in_M9	Eco_in_LB	Eco_Pr	Eco_Rh		
1	5'-terminal flanking region of 16S rRNA genes	*rrsH*	+	223409	223770	–		223656*	223656	++	ACTCGAAGATACgg**t**gtgggc
							**223657**			++	AGATACGGATTCt**g**tgggcactcga
		rrsG	-	2731158	2731599	–	2731248*	2731253		+-	agatacggatt**c**tGTGGGCACTCGA
							**2731271***	2731272	2731272	- -	AGATACGGATTCt**g**tgggcactcga
							2731325			+ -	cgcgc**c**gaaggcgtaTTATGCACACCC
		rrsD	-	3428763	3429235	–	3428853	3428853*		+ -	agatacggatt**c**tGTGGGCACTCGA
							**3428876**	3428877*	3428877	- -	AGATACGGATTCt**g**tgggcactcga
		rrsC	+	3941328	3941807	–	**3941694***	3941693	3941693	+ +	AGATACGGATTCt**g**tgggcactcg
							3941717*			- +	agatacggatt**c**tGTGGGCACTCGA
		rrsA	+	4035154	4035530	–	4035363			- +	cgcgc**c**gaaggcgtaTTATGCACACCC
							**4035417***	**4035416**	4035416	+ +	AGATACGGATTCt**g**tgggcactcga
							4035440			- +	agatacggatt**c**tGTGGGCACTCGA
		rrsB	+	4166286	4166658	–	4166491			- +	cgcgc**c**gaaggcgtaTTATGCACACCC
							**4166545***	4166544	4166544	+ +	AGATACGGATTCt**g**tgggcactcga
							4166568			- +	agatacggatt**c**tGTGGGCACTCGA
		rrsE	+	4207533	4208146	–	**4208033**	**4208032**	4208032*	++	ACTCGAAGATACgg**t**gtgggc
							4208056			- +	agatacggatt**c**tGTGGGCACTCGA
2	Coding sequence of 16S rRNA gene	*rrsH*	+	223771	225312	223782			223772*	+ +	TGATCATGGCTCagattgaaaattgaagagt
									223802*	+ +	tgatcatggctcagattgaaAATTGAAGAGTT
						223823				- +	aacgctggcggcaggcctaac**a**tttgatcaTGGCTCAGATTG
									224079	- +	TGAGACACGGTCcagac**a**gccacactggaac
									224108	+ +	tgagacacggtccag**a**cagCCACACTGGAAC
								224291		+ +	GAGGGTGCAAGCgttaatcggaatta**g**cagccgcggtaatacg
								224332		- +	gagggtgcaagcgttaatcggaat**t**agcagcCGCGGTAATACG
								224363		- +	ggtttg**t**ttaatcggaattactgggcgtaaaGCGCACGCAGGC
							224475			- +	gtaga**g**gggggtagaattccaggtgtaGCGGTGAAATGC
						224527				+ +	TGGGGAGCAAACa**t**caggtgcgaaagcg
						224554				- +	tggggagcaaacatcaGGTGCGAAAGCG
							225016			+ +	GAGAAGCGACCTcgcgagagcaagc**a**tacaaa
						225080				- +	ggattg**g**GCGTCGTAGTCC

	Coding sequence of 16S rRNA gene	*rrsG*	-	2729616	2731157	2729848				+ -	ggattggGCGTCGTAGTCC
							2729912			- -	GAGAAGCGACCTcgcgagagcaagcatacaaa
						2730374				+ -	tggggagcaaacatcaGGTGCGAAAGCG
						2730401				- -	TGGGGAGCAAACatcaggtgcgaaagcg
							2730453			+ -	gtagaggggggtagaattccaggtgtaGCGGTGAAATGC
								2730637		- -	GAGGGTGCAAGCgttaatcggaattagcagccgcggtaatacg
									2730820	+ -	tgagacacggtccagacagCCACACTGGAAC
									2730849	- -	TGAGACACGGTCcagacagccacactggaac
						2731105				+ -	gttccagtgtggctgtctgGACCGTGTCTCA
						2731146*			2731156	- -	AACGCTGGCGGCaggcctaacatttgatcatggctcagattg
	Coding sequence of 16S rRNA gene	*rrsD*	-	3427221	3428762	3427453				+−	ggattggGCGTCGTAGTCC
							3427517			−−	GAGAAGCGACCTcgcgagagcaagcatacaaa
						3427979				+−	tggggagcaaacatcaGGTGCGAAAGCG
						3428006				−−	TGGGGAGCAAACatcaggtgcgaaagcg
							3428058			+−	gtagaggggggtagaattccaggtgtaGCGGTGAAATGC
								3428242		−−	GAGGGTGCAAGCgttaatcggaattagcagccgcggtaatacg
									3428425	+−	tgagacacggtccagacagCCACACTGGAAC
									3428454	−−	TGAGACACGGTCcagacagccacactggaac
						3428710				+−	aacgctggcggcaggcctaacatttgatcaTGGCTCAGATTG
						3428751			3428761*	−−	AACGCTGGCGGCaggcctaacatttgatcatggctcagattg
	Coding sequence of 16S rRNA gene	*rrsC*	+	3941808	3943349	**3941819***			**3941809**	+ +	TGATCATGGCTCagattgaaaattgaagagtt
									3941839	- +	tgatcatggctcagattgaaAATTGAAGAGTT
						3941860				- +	aacgctggcggcaggcctaacAtttgatcaTGGCTCAGATTG
									3942116	+ +	TGAGACACGGTCcagacagccacactggaac
									3942145	- +	tgagacacggtccagacagCCACACTGGAAC
								3942328		+ +	GAGGGTGCAAGCgttaatcggaattagcagccgcggtaatacg
							3942369			- +	gagggtgcaagcgttaatcggaattagcagcCGCGGTAATACG
								3942400		- +	ggtttgattaatcggaattactgggcgtaaaGCGCACGCAGGC
							3942512			- +	gtagaggggggtagaattccaggtgtaGCGGTGAAATGC
						3942564				+ +	TGGGGAGCAAACatcaggtgcgaaagcg
						3942591				- +	tggggagcaaacatcaGGTGCGAAAGCG
							3943053			+ +	GAGAAGCGACCTcgcgagagcaagcatacaaa
						3943117				- +	ggattggGCGTCGTAGTCC
						3943344				+ +	GTTCTTTGCAGTgctcacacatccttaccttaaagaagc

	Coding sequence of 16S rRNA gene	*rrsA*	+	4035531	4037072	4035542*			4035532	++	AACGCTGGCGGCaggcctaacatttgatcatggctcagattg
									4035562	- +	tgatcatggctcagattgaaAATTGAAGAGTT
						4035583				- +	aacgctggcggcaggcctaacatttgatcaTGGCTCAGATTG
								4035562		- +	tgatcatggctcagattgaaAATTGAAGAGTT
								4035583		- +	aacgctggcggcaggcctaacatttgatcaTGGCTCAGATTG
									4035839	++	TGAGACACGGTCcagacagccacactggaac
									4035868	- +	tgagacacggtccagacagCCACACTGGAAC
								4036051		++	GAGGGTGCAAGCgttaatcggaattagcagccgcggtaatacg
								4036092		- +	gagggtgcaagcgttaatcggaattagcagcCGCGGTAATACG
								4036123		- +	ggtttgtttaatcggaattactgggcgtaaaGCGCACGCAGGC
							4036235			- +	gtagagggggggtagaattccaggtgtaGCGGTGAAATGC
						4036287				++	TGGGGAGCAAACatcaggtgcgaaagcg
								4036314		- +	tggggagcaaacatcaGGTGCGAAAGCG
							4036776			++	GAGAAGCGACCTcgcgagagcaagcatacaaa
						4036840				- +	ggattggGCGTCGTAGTCC
	Coding sequence of 16S rRNA gene	*rrsB*	+	4166659	4168200	4166670			4166660*	++	TGATCATGGCTCagattgaaaattgaagagtt
								4166690		- +	tgatcatggctcagattgaaAATTGAAGAGTT
						4166711				- +	aacgctggcggcaggcctaacatttgatcaTGGCTCAGATTG
									4166967	++	TGAGACACGGTCcagacagccacactggaac
									4166996	- +	tgagacacggtccagacagCCACACTGGAAC
								4167179		++	GAGGGTGCAAGCgttaatcggaattagcagccgcggtaatacg
								4167220		- +	gagggtgcaagcgttaatcggaattagcagcCGCGGTAATACG
								4167251		- +	ggtttgtttaatcggaattactgggcgtaaaGCGCACGCAGGC
							4167363			- +	gtagagggggggtagaattccaggtgtaGCGGTGAAATGC
						4167415				++	TGGGGAGCAAACatcaggtgcgaaagcg
						4167442				- +	tggggagcaaacatcaGGTGCGAAAGCG
							4167904			++	GAGAAGCGACCTcgcgagagcaagcatacaaa
						4167968				- +	ggattggGCGTCGTAGTCC
		rrsE	+	4208147	4209688	4208158*			4208148	++	AACGCTGGCGGCaggcctaacatttgatcatggctcagattg
									4208178	- +	tgatcatggctcagattgaaAATTGAAGAGTT
						4208199				- +	aacgctggcggcaggcctaacatttgatcaTGGCTCAGATTG
									4208455	++	TGAGACACGGTCcagacagccacactggaac
									4208484	- +	tgagacacggtccagacagCCACACTGGAAC
								4208667		++	GAGGGTGCAAGCgttaatcggaattagcagccgcggtaatacg

								4208708		- +	gagggtgcaagcgttaatcggaat**t**agcagcCGCGGTAATACG
								4208739		- +	ggtttg**t**ttaatcggaattactgggcgtaaaGCGCACGCAGGC
							4208851			- +	gtaga**g**gggggtagaattccaggtgtaGCGGTGAAATGC
						4208903				+ +	TGGGGAGCAAACa**t**caggtgcgaaagcg
						4208930				- +	tggggagcaaac**a**tcaGGTGCGAAAGCG
							4209392			+ +	GAGAAGCGACCTcgcgagagcaagc**a**tacaaa
						4209456				- +	ggattg**g**GCGTCGTAGTCC
						4209683				+ +	GTTCTTTGCAGTgctcacaca**t**ccttaccttaaagaagc
3	Coding sequence of 23S rRNA gene	*rrlH*	+	225759	228662		225759			+ +	CTAAGCGTACACggtggatg**g**gttaagcga
						225804				+ +	TAATCTGCGATAag**g**cgatgaaggacgtgc
								225831		- +	taatctgcgat**a**aggcgaTGAAGGACGTGC
								225913		+ +	ATCCATAGGTTAatgaggcgaaccgggg**a**actga
						226267				- +	aaaagaa**c**ccccggCGAGGGGAGTGA
									226379	+ +	GGAAACCGAGTCt**a**gccgaag
									226562	- +	gggagat**a**GCCAATCAAACC
						226775				+ +	GAAACGATGTGGga**g**ttaagtgg
						227245				- +	tggttt**t**cgggcgacggttgtcccggtttAAGCGTGTAGGC
									227992	+ +	AGTTTGACTGGGgcggtctcct**g**gtgggt
						228031				+ +	GAGCACGAAGGT**a**acggag
						228047				- +	acgaa**g**ctcctaaagagTAACGGAGGAGC
							228049			+ +	ACATCAGGAGGTtagtg**t**tggctaatcctggtcgg
						228074				+ +	TGCAATGGCATAagccag**g**aggttag
							228082			- +	acatcaggaggttag**t**gttggctAATCCTGGTCGG
								228094		+ +	GCTTGACTGCGAgcgtgacggcgcgagcagg**a**gcca
							228258			- +	gtttggcacc**t**gttcatATCGACGGCGGT
						228316				+ +	AGCTGGGTTTAGa**c**catttaaagtggtacgcg
						228347				- +	agctgggtttagaccatttaAAGTGGTACGCG
						228444				- +	actg**g**ggggggctgctcctagtacgagaggaccggAGTGGACGCATC
							228621			+ +	GTACTAATGAACcgtgaggcttaacctt**c**gttgagctaaccg
								228638	**228635***	+ +	AACCGTGAGGCTtaacct**g**tactaatg
									228644	+ +	TAACCTTACAACgcc**a**accgtgaggct
							228662		228661*	- +	gtactaatgaaccgtgaggcttaacct**t**cgTTGAGCTAACCG

4	Coding sequence of 5S rRNA gene	*rrfH*	+	228756	228875	**228760**	**228760**			++	CGGTGGTCCCACctgacctggcggccgtagcg
						228768*	228772			++	GGTGGTCCCACCtgaccgtagcgc
						228773				- +	tagcgatttGCCTGGCGGCCG
							**228773**			++	GGTCCCACCTGAccccatgccgaacgcggt
							228777			++	CAGAAGTGAAACtggtcccacctgaccccatgccgaact
							228779			++	CCCCATGCCGAActcaggtcccacctga
						228780				- +	gcggtggtctGGCGGCCGTAGC
							228782			++	CCATGCCGAACTcaccacctgacc
						228789	**228789**			- +	cccacctgaacctggcggccgTAGCGCGGTGGT
							228791			- +	cccacctgaccgcggccgTAGCGCGGTGGT
							**228792**			++	CAGAAGTGAAACgccgtagcgccgatggtagtgtgccatgccgaact
							**228792**			- +	cacctgaccccctggcggccgtaGCGCGGTGGTCC
							**228793**			++	ACTCAGAAGTGAaacgccgtagcgccgatggtagtgtcatgccga
							**228793**			- +	cacctgaccccctaGCGCGGTGGTCC
							228794			++	AGAAGTGAAACGccgtagcgccgatggtagtgatgccgaactc
							**228794**			- +	ccacctgaccccatgcctggcggccgtAGCGCGGTGGTC
							**228795**			++	GAAGTGAAACGCcgtagcgccgatggttgccgaactca
							228796			++	GAAGTGAAACGCcgtagcgccgatgggccgaactca
							228798			++	GAAGTGAAACGCcgtagcgccgatggtagtgcgaactca
							228799			++	AAGTGAAACGCCgtagcgccgatggtagtggaactcag
						228800	228801*			++	GCCGATGGTAGTGactcagaagtgaaacgccgtagc
						228803	**228803**			++	GCCGATGGTAGTgtgtcagaagtgaaacgccgtagc
						228804*	**228804**			++	GCCGATGGTAGTgtcagaagtgaaacgccgtagc
						228805	228805			++	GGTAGTGTGGGGtctcccagaagtgaaacgccgtagcgccgat
							228805			- +	tcggcgctacggcgtttcacttctgggagacCCCACACTACCA
						**228807**	228806*			++	TGGTAGTGTGGGgtctccccagaagtgaaacgccgtagcgccga
						228812	228811*			++	GCCGATGGTAGTgtggggtgaaacgccgtagc
						228814	228815*			- +	cagaagtgaaactggtcccacctgaccCCATGCCGAACT
							228817			++	CGATGGTAGTGTggccgtagcgc
	Coding sequence of 5S rRNA gene	*rrfH*	+	228756	228875	228821	228818*			++	CGCCGATGGTAGtgtgcgtag
						228823	228819*			- +	cgccgcatgccgaacTCAGAAGTGAAA
						228824	**228825***			- +	gtagcgcactcagAAGTGAAACGCC
						228825	**228826***			++	CGAGAGTAGGGAactgcccgatggtagtgtggggtctccccatg
						**228825**				- +	gtagcgcactcagAAGTGAAACGCC

						228831	**228827***			++	ATGCGAGAGTAGggaactgccgatggtagtgtggggtctcccc
						228829*	228828			- +	gcgccgatcgaactcagaagTGAAACGCCGTA<br>cgatgagaagtgaaACGCCGTAGCGC
						**228830**	228830			- +	
						**228831***				- +	ccgatggcagaagtgaAACGCCGTAGCG<br>gccgatggtaccatgccgaactcagaagtgAAACGCCGTAGC
						**228833***	228833			- +	
						**228835***	228835			- +	cgatggtagtaactcagaagtgaaACGCCGTAGCGC<br>gaaacgccgtagcgccgatggtagtgccatgccGAACTCAGAAGT
						**228836**	**228836***			- +	
						**228837***	**228837**			- +	gccgatggtagtgttcagaagtgAAACGCCGTAGC
							**228838**			++	AGAGTAGGGAACtgccggggtctccccatgcg<br>cagaagtgaaacgccgtagcgccgatggtagtgtgCCATGCCGAACT
						228839	**228838**			- +	
							228839			++	GAGAGTAGGGAActgccagggggtctccccatgc<br>CGAGAGTAGGGAActgccagggggtctccccatg
							**228840**			++	
							**228840**			- +	gccgatggtagtgtggggtgAAACGCCGTAGC
							**228841**			++	TAGGGAACTGCCagggtctccccatgcgagag<br>GAGTAGGGAACTgccaggtctccccatgcga
							**228842**			++	
						228844	**228844**		228844*	++	GAGAGTAGGGAActgccaggcatctccccatgc
						228845				- +	gggtctcccGATGGTAGTGTG
						228846				- +	tggtagtgtggggtctcccagaagtgaaacgCCGTAGCGCCGA<br>TAGGGAACTGCCcccatgcgagag
							**228846**			++	
							**228847**			- +	tggtagtgtggggtctccccagaagtgaaacgCCGTAGCGCCGA
						228849				++	AGAGTAGGGAACatgcg<br>GGAACTGCCAGGcatgcgagagtag
						228851				++	
						228860				- +	agagtaggggggTCTCCCCATGCG
							228866*			- +	atgcgagagtagggaactgccgatggtagtgTGGGGTCTCCCC<br>gaactgccaTGCGAGAGTAGG<br>cgagagtagggaactgcccgatggtagtgtggGGTCTCCCCATG<br>tgcgagagtagggaactgccGGGGTCTCCCCA<br>agagtagggaactgccaggggTCTCCCCATGCG
						**228867***	**228867**			- +	
						228868	**228868***			- +	
							**228869**			- +	
							**228870***			- +	
							228871			- +	gagagtagggaactgccaggctgggGTCTCCCCATGC<br>ggaactgccaggcATGCGAGAGTAG<br>gagagtagggaactgccaggctgggGTCTCCCCATGC<br>agggaactgccaggcatctccCCATGCGAGAGT
						228872				- +	
		rrfH	+	228756	228875	228873				- +	
							**228874**			- +	
							228875			- +	aggcatccatgcgagagTAGGGAACTGCC

5	tRNA	*glnW*	-	696756	696830	696792	696830*		696830	- -	GCCAAGCGGTAAggcaccggtttttgata**t**ggggtatc
6	tRNA	*valW*	+	1746516	1746592		1746516			+ +	ACCACCTTGACAtggtg**c**gtccgtagctcagttggttagagc
						1746517*	1746517	1746517		+ +	
							1746555			+ -	accaccttgacatg**g**tgcgtccgtagctcaGTTGGTTAGAGC
						1746556	1746556	1746556*		+ -	
7	sRNA	*ryeA*	+	1923066	1923337				1923097	+ +	AAAAGAGACCGAaca**a**acagata
						1923125				+ -	gaatacag**g**caagagccaTTTCCCTGGACC
								1923308		+ +	GAACTGAGGCGGtttt**c**tggtgaaa
8	tRNA	*glyW*	-	1992042	1992117		1992042			+ -	ttcccgctcc**a**cgcgagTTCGAGTCTCGT
						1992080*	1992080	1992081		- -	cacgaccttgcca**a**gcgggaatagctCAGTTGGTAGAG
						1992116	1992116	1992117*		- -	GACCTTGCCAAGgt**g**cgggaatagctcagttggtagagcac
		glyV	+	4392360		**4392361**	**4392361**	**4392360***		+ +	GACCTTGCCAAGgt**g**cgggaatagctcagttggtagagcac<br>acgacctt**g**cgcgggaatagctcAGTTGGTAGAGC
					4392435			4392393*		- +	
						4392397*	4392397	4392396		- +	cacgaccttgcca**a**gcgggaatagctCAGTTGGTAGAG
								4392398		++	TCGCGAGTTCGAgtctcgtttcccgctccaaa**g**gtcgggg
								4392400*		- +	gaccttgccaagg**t**gcgggaatagctcagTTGGTAGAGCAC
							4392435			- +	ttcccgctcc**a**cgcgagTTCGAGTCTCGT
	tRNA	*glyX*	+	4392472	4392547	4392473*	4392473	4392472		+ +	CACGACCTTGCCaag**c**gggaatagctcagttggtagag<br>cgacctt**g**ccaagcgggaatagctcaGTTGGTAGAGCA<br>acgaccttg**c**agcgggaatagctcAGTTGGTAGAGC
						4392505	4392505*	4392506		- +	
						4392506*		4392507		- +	
								4392510*		+ +	TCGCGAGTTCGAgtctcgtttcccgctccaaa**g**gtcgggg
								4392512		- +	gaccttgccaagg**t**gcgggaatagctcagTTGGTAGAGCAC
							4392547			- +	ttcccgctcc**a**cgcgagTTCGAGTCTCGT
		glyY	+	4392583	4392658	4392584	4392584*	4392583		+ +	CACGACCTTGCCaag**c**gggaatagctcagttggtagag
						4392616	4392617	4392617		- +	cgacctt**g**ccaagcgggaatagctcaGTTGGTAGAGCA
						4392617		4392618		- +	acgaccttg**c**agcgggaatagctcAGTTGGTAGAGC
								4392621		+ +	TCGCGAGTTCGAgtctcgtttcccgctccaaa**g**gtcgggg
								4392623*		- +	gaccttgccaagg**t**gcgggaatagctcagTTGGTAGAGCAC
							4392658			- +	ttcccgctcc**a**cgcgagTTCGAGTCTCGT
9	tRNA	*asnT*	+	2044549	2044624	2044552		2044561*		+ +	GGCGGACTGTTAatcc**c**agtcggtagaac
							2044577			+ +	TTCGAGTCCAGTcag**g**gactgttaatccgtatgtcactgg

						2044586				- +	gaacggcggactgttaatcctctgtaGTTCAGTCGGTA
								2044589		+ +	CAGTCAGAGGAGccacgtatgtcactggttcgagtc<br>ggcggactgttaatcccAGTCGGTAGAAC
							2044589			- +	
						2044591				+ +	TCGAGTCCAGTCagtatgtcactggt<br>TCCAGTCAGAGGggttcgag<br>TCCAGTCAGAGGttcgag
						2044600				+ +	
						2044602				+ +	
						2044615	2044616*			- +	ttcgagtccagtcagggactgttaatccGTATGTCACTGG<br>cagtcagacgtatgtcaCTGGTTCGAGTC
							2044617			- +	
						2044621				- +	aggagctggttcGAGTCCAGTCAG<br>gaggagccattaatccgtatgtcactggttCGAGTCCAGTCA
						**2044624**	2044624			- +	
	tRNA	*asnW*	-	2058027	2058102	**2058027***	2058027			+ -	gaggagccattaatccgtatgtcactggttCGAGTCCAGTCA
						2058030	2058034*			+ -	cagtcagacgtatgtcaCTGGTTCGAGTC<br>ttcgagtccagtcagggactgttaatccGTATGTCACTGG
						2058036	2058035*			+ -	
						2058049				- -	TCCAGTCAGAGGttcgag
						2058051				- -	TCCAGTCAGAGGggttcgag
						2058060	2058062*			- -	CAGTCAGAGGAGccacgtatgtcactggttcgagtc
						2058065				+ -	gaacggcggactgttaatcctctgtaGTTCAGTCGGTA
							2058074			- -	TTCGAGTCCAGTcaggactgttaatccgtatgtcactgg<br>GGCGGACTGTTAatcccagtcggtagaac
								2058090		- -	
						2058099				- -	GAACGGCGGACTgttaatcctctgtagttcagtcggta
	tRNA	*asnU*	+	2059851	2059926	2059854				+ +	GAACGGCGGACTgttaatcctctgtagttcagtcggta<br>GGCGGACTGTTAatcccagtcggtagaac
								2059863*		+ +	
							2059879			+ +	TTCGAGTCCAGTcaggactgttaatccgtatgtcactgg<br>gaacggcggactgttaatcctctgtaGTTCAGTCGGTA
						2059888				- +	
							2059891			+ +	CAGTCAGAGGAGccacgtatgtcactggttcgagtc
	tRNA	*asnU*	+	2059851	2059926			2059891*		- +	ggcggactgttaatcccAGTCGGTAGAAC
						2059893				+ +	TCGAGTCCAGTCagtatgtcactggt
						2059902				+ +	TCCAGTCAGAGGggttcgag<br>TCCAGTCAGAGGttcgag*
						2059904				+ +	
						2059917	2059918*			- +	ttcgagtccagtcagggactgttaatccGTATGTCACTGG
						2059923	2059919*			- +	cagtcagacgtatgtcaCTGGTTCGAGTC
						**2059926**	2059926			- +	aggagccagtatgtcactggttcGAGTCCAGTCAG
	tRNA	*asnV*	+	2062260	2062335	2062263				+ +	GAACGGCGGACTgttaatcctctgtagttcagtcggta

								2062272		+ +	GGCGGACTGTTAatcccagtcggtagaac
							2062288			+ +	TTCGAGTCCAGTcagggactgttaatccgtatgtcactgg<br>gaacggcggactgttaatcctctgtaGTTCAGTCGGTA
						2062297				- +	
								2062300		- +	ggcggactgttaatcccAGTCGGTAGAAC
						2062302	2062300*			+ +	CAGTCAGAGGAGccacgtatgtcactggttcgagtc
						2062311				+ +	TCCAGTCAGAGGggttcgag<br>TCCAGTCAGAGGttcgag
						2062313				+ +	
						2062326	2062327*			- +	ttcgagtccagtcagggactgttaatccGTATGTCACTGG
						2062332	2062328*			- +	cagtcagacgtatgtcaCTGGTTCGAGTC
						2062335*	2062335			- +	gaggagccattaatccgtatgtcactggttCGAGTCCAGTCA
10						2518043				+ -	tagctccaccatgcaagaggtcagcggTTCGATCCCGCT
	tRNA	*alaX*	-	2518041	2518116			2518052		+ -	agaggtcagcggttcgatcccgctttGCATGGCATGCA
							2518116	2518116		- -	GCTTGCATGGCAggggctatagctcagctgggagagc
11							2729426			- -	TCACGGCGGTAAcaggcccaggacaccgcccctt
	tRNA	*gltW*	-	2729369	2729444		2729426			+ -	tagaggcGTCCCCTTCGTC
						2729443	2729435		2729444*	- -	TAGAGGCCCAGGacaccgccccttgtccccttcgtc
						**3943436**			3943435*	+ +	TAGAGGCCCAGGacaccgccccttgtccccttcgtc
							3943444			+ +	AGGACACCGCCCtttcacggcggtaacgtctagaggccc
							3943452			- +	tagaggcGTCCCCTTCGTC
							3943453			+ +	TCACGGCGGTAAcaggcccaggacaccgcccctt<br>GGTAACAGGGGTtcgaatcccctttcacggc
	tRNA	*gltU*	+	3943435	3943510		3943469			+ +	
									3943469	- +	tagaggcccaggacaccgcccctGTCCCCTTCGTC
							3943481			- +	aggacaccgccctttcacggcggtaacGTCTAGAGGCCC
							3943482			- +	tcacggcggtaacaggcccagGACACCGCCCTT
							3943495			- +	ggtaacaggggttcgaatcCCCTTTCACGGC<br>gggacgccacacggcggtaacaggggttCGAATCCCCTAG
							3943510			- +	
						4168373			4168372*	+ +	TAGAGGCCCAGGacaccgccccttgtccccttcgtc
							4168381			+ +	AGGACACCGCCCtttcacggcggtaacgtctagaggccc
	tRNA	*gltT*	+	4168372	4168447		4168390			- +	TCACGGCGGTAAcaggcccaggacaccgcccctt<br>tagaggcccaggacaccgccccttGTCCCCTTCGTC
									4168405	- +	
							4168406			+ +	GGTAACAGGGGTtcgaatcccctttcacggc
							4168418			- +	aggacaccgccctttcacggcggtaacGTCTAGAGGCCC

							4168419			- +	tcacggcggtaacaggcccagGACACCGCCCTT
							4168432**			- +	ggtaacaggggttcgaatcCCCTTTCACGGC
							4168447			- +	gggacgccacacggcggtaacaggggttCGAATCCCCTAG
	tRNA	*gltV*	+	4209774	4209849	**4209775**			4209774	+ +	TAGAGGCCCAGGacaccgcccttgtccccttcgtc
							4209783			+ +	AGGACACCGCCCtttcacggcggtaacgtctagaggccc
							4209791			- +	tagaggcGTCCCCTTCGTC
							4209792			+ +	TCACGGCGGTAAcaggcccaggacaccgccctt
							4209808			+ +	GGTAACAGGGGTtcgaatccccttcacggc
									4209808	- +	tagaggcccaggacaccgcccttGTCCCCTTCGTC
							4209820			- +	tcacggcggtaacaggcccagGACACCGCCCTT
							4209821			- +	ggtaacaggggttcgaatcCCCTTTCACGGC
							4209834			- +	ggtaacaggggttcgaatcCCCTTTCACGGC
							4209849			- +	gggacgccacacggcggtaacaggggttCGAATCCCCTAG
12	sRNA	*ryfD*	-	2734153	2734295		2734153			+-	cgtctggatcgtcttactgttatgGGAGGAGTTATG
							2734188*		2734187	- -	CGTCTGGATCGTcttactgttatgggaggagttatg
								2734196*		- -	GAGGAGTTATGCgtctggatcgtcttactttgatccgttatgg
							2734207			+-	gatgacctcatttaatctcGAATAATTGAGG
							2734237			- -	GATGACCTCATTtaatctcgaataattgagg
13	sRNA	*ssrS*	+	3055983	3056165	3055978*	3055975			+ +	ATTTCTCTGAGAtgttcgcaagcgggccagacaaa
						3055983				+ +	GTTCGCAAGCGGgccagtcccctatttctctgagat
							3056005*			- +	tctgagatgttcgcaagcgaAGACAAAATTTC
						3056018				- +	gttcgcaagcgggccagtcccctaTTTCTCTGAGAT
							3056043			- +	acaagaatgagccgATATTTCATACC
						3056117				+ +	ATCTCGGAGATTcctgaaccaagggttcaagggttacagcctgcggcggc
						3056120				+ +	TACAGCCTGCGGcggcatctcggagattcccaccaagggttcaagggt
								3056136		+ +	GCCTGCGGCGGCatctcggagattccttaca
							**3056138**	3056138	3056138*	+ +	ATCTCGGAGATTcccttacagcctgcggcggc
						3056166*	3056166			- +	atctcggagattcctgaaccaagggttcaagggttacaGCCTGCGGCGGC
						3056167*	**3056167**		3056167	- +	tacagcctgcggcggcatctcggagattcccaccaaGGGTTCAAGGGT
14	tRNA	*aspT*	+	3946872	3946948		3946867			++	GGAGCGGTAGTTcagttcggt
						3946904				- +	acctgccggagcggtagttcaGTCGGTTAGAAT
						3946927				++	CGTCCGTTCCGCcatcgagtcc

							3946948*	3946948		- +	cgttccgccatcacgcagggggtcgcgggtTCGAGTCCCGTC
15							3982509*			++	CCTGGATTGTGAttggtggctatagctcagttggtagagc<br>cctggattgtggtggctatagctcAGTTGGTAGAGC
							3982542			- +	
							3982547			- +	cctggattgtgattggtggctatagctcAGTTGGTAGAGC<br>gattccagtagctcagttggtagaGCCCTGGATTGT
	tRNA	*hisR*	+	3982509	3982585				3982552	- +	
									3982561*	++	ATCCCATTAGCCaccccaggttcga<br>ttcgaaattgtgattccAGTTGTCGTGGG<br>ccattacagttgtcgTGGGTTCGAATC
						3982568	3982568			- +	
							3982576*			- +	
16						3982606	3982606	3982606*		++	GACGCGCTAGCTtcaggtggcgaaggtggcggaattggta<br>GCGGAATTGGTagacgcgcgaaggt
						3982608*				++	
						3982637				- +	ggaattggtagacgcgctagcTGCGAAGGTGGC<br>cgctagcttgcgaaggtggcggAATTGGTAGACG<br>gacgcgctagcttcaggtggcgaaggtgGCGGAATTGGTA
									3982638	- +	
	tRNA	*leuT*	+	3982606	3982692			3982645*		- +	
							3982655			++	GGGTTCAAGTCCttacggacgtgg<br>CCCCCTCGCACCagggttcaagtccc
							3982666			++	
						3982674	3982674*			- +	gggttcaagtccTTACGGACGTGG**<br>ccccctcgcaccaggGGTTCAAGTCCC
							3982692			- +	
							4606079			+ -	ccccctcgcaccaggGGTTCAAGTCCC
						4606097	4606097*			+ -	gggttcaagtccTTACGGACGTGG
							4606105			- -	CCCCCTCGCACCagggttcaagtccc<br>GGGTTCAAGTCCttacggacgtgg
	tRNA	*leuV*	-	4606079	4606165		4606116			- -	
						4606134*				+ -	ggaattggtagacgcgctagcTGCGAAGGTGGC<br>GGCGGAATTGGTagacgcgcgaaggt<br>GGAATTGGTAGAcgcgctagctgcgaaggtggc
							4606163			- -	
						4606165*	4606165	4606165		- -	
							4606200			+ -	ccccctcgcaccaggGGTTCAAGTCCC<br>cccccctgttcttacggacgtggGGGTTCAAGTCC<br>ccccctgttcttacggacgtggGGGTTCAAGTCC
							**4606208**			+ -	
							**4606209**			+ -	
	tRNA	*leuP*	-	4606200	4606286		**4606214**			+ -	ggttcaagtccctagtgttctTACGGACGTGGG
						4606215	4606215			+ -	
							4606226			- -	CCCCCTCGCACCagggttcaagtccc
							4606237			- -	GGGTTCAAGTCCttacggacgtgg<br>GGGTTCAAGTCCtgttcttacggacgtgg
							4606242			- -	

							4606284			- -	GGCGGAATTGGTagacgcgcgaaggt<br>GACGCGCTAGCTtcaggtg**g**cgaaggtggcggaattggta
						4606286	4606286*	4606286		- -	
	tRNA	*leuQ*	-	4606315	4606401		4606315			+ -	cccccctcgcacc**a**ggGGTTCAAGTCCC
						4606333	4606333			+ -	gggttca**a**gtccTTACGGACGTGG
							4606341			- -	CCCCCTCGCACCa**g**gggttcaagtccc
							4606352			- -	GGGTTCAAGTCC**t**tacggacgtgg
						4606370				+ -	ggaattggtagacgcgctag**c**TGCGAAGGTGGC<br>GGCGGAATTGGTagacgcgc**g**aaggt<br>GACGCGCTAGCTtcaggtggcgaaggtggcggaattggta
							4606399			- -	
						4606401	**4606401**	4606401*		- -	
17	Inter-genic region	*fimA-fimI*	+	4543664	4543727	**4543667**	4543666*			+ +	GGTTCAGGGACGtcattacgggcagggatgccc**t**accca
							4543668			+ +	ATTACGGGCAGGgatgcccaccct**c**ccaggttcagggacgtc
							4543671	4543671*		+ +	ATTACGGGCAGGgatgcccaccct**a**ggttcagggacgtc
						**4543673***	**4543673**	4543673		+ +	ACGTCATTACGGgcagggatgcccaccc**g**ttcaggg
						**4543674**				+ +	ATTACGGGCAGGgatgcccacc**t**tcagggacgtc
						**4543675**	4543675*			+ +	ACGTCATTACGGgcagggatgcccaccctt**t**caggg
						**4543676**	4543676	4543676*		+ +	TCATTACGGGCAgggatgcccaccctt**c**agggacg
						4543706*	4543704			- +	acgtcattacgggcagggatgccca**c**cctacCCAGGTTCAGGG
						4543707				- +	agggatgcccacc**c**attacgggcagggatgcccacCTTCAGGGACGTC
						**4543708**	**4543708**			- +	agggatgcccacc**c**acccaggttcagggacGTCATTACGGGC
						**4543709***	**4543709**	**4543709**		- +	attacgggcagggatgcccaccc**t**cccaggTTCAGGGACGTC
