# Supplementary material for: The Fate and Functionality of Alien tRNA Fragments in Culturing Medium and Cells of Escherichia coli
Source: Int J Mol Sci. 2023 Aug 19;24(16):12960. doi: 10.3390/ijms241612960 (PMC10455298; doi:10.3390/ijms241612960)
Supplement: Supplementary file 1 [file ijms-24-12960-s001.zip › Table_S3.pdf]

Table S3 Genomic origins of permuted oligonucleotides found in extracellular milieu from at least two of the four types of experiments

- Indicated Genomic positions correspond to the ligation sites (LS) in the genomic loci as shown in Figure 6e.
- Depending on the scanned strand and the localization of the LS found in the oligos, four types of rearrangements are possible as described in the Legend of the Table S2. Their codes are indicated in the last column of the Table.
- Positions with multiple occurrences are marked in bold, and those that turned out to be the same in different experiments are similarly colored.
- All permutation events found in at least 2 types of experiments are listed, including those found in RNA products of paralogous genes (16S rRNA and 23S rRNA) with a similar pattern of their distribution.
- Examples, illustrating rearrangement types are shown in Table S2. The codes used to denote the four types of rearrangements are as follows:

  anchor 12-mer found upon **top** strand scanning and matched to the **top** strand (++);
  top strand scanning, **bottom** strand matching (+-);
  bottom strand scanning, **bottom** strand matching (--);
  bottom strand scanning, **top** strand matching (-+)
- Genomic coordinates of ligation sites of permuted transcripts also present inside bacterial cells (Table S2) are underlined.
- Coordinates of permuted fragments registered in multiple copies are shown in bold.

N	Type of genomic region	Ge-ne(s)	Str	Borders of gene		Left borders of loci whose RNA products underwent permutation (ligation sites)				Co-de	Examples of oligos, showing uni- and bidirectional permutation, as well as predominant location of ligation sites (marked red).
				Left	Right	Eco_exo_M9	Eco_out_LB	Eco_Prev	Eco_Rhod		
1	Coding sequence of 16S rRNA gene	*rrsH*	+	223771	225312	223828				- +	taacacatg**c**tgaacgcTGGCGGCAGGCC
						224056				++	AGGATGACCAGCcacactg**c**tagctggtctgag
						224088				- +	aggatgaccagccacact**g**ctAGCTGGTCTGAG
						224131				- +	cagtgg**g**ctgagacacggtccagactccTACGGGAGGCAG
						224134				++	TGCAGCCATGCCg**a**tattgcacaatgggcgcaagcctga
						224161*				++	TGTATGAAGAAGgcct**g**cagccatgccgcg
						224172*				- +	tgcagccatgcc**g**atattgcacaatggGCGCAAGCCTGA
						224189				- +	tgtatgaagaaggc**c**tgcAGCCATGCCGCG
						224212				- +	ttca**g**cttcgggTTGTAAAGTACT
						224303*				++	CGTTAATCGGAAttactgggc**a**tacggagggtgcaag

						224339*				- +	cgttaatcggaattactgggcatacGGAGGGTGCAAG
						224448*				++	ATGCGTAGAGATctggaggaattccaggtgtagcggtgaa
						224483*				- +	atgcgtagagatctggaggaattccaggTGTAGCGGTGAA
									224543*	++	TTAGATACCCTGgtagtccacgccgtaaacgaggggagcaaacagga
									224589*	- +	ttagataccctggtagtccacgccgtaaacgaagggGAGCAAACAGGA
						224870				++	GCAACCCTTATCcaacgagc
						225012				- +	tacaatggccagggCTACACACGTGC
						225056				++	gCGTCGTAGTCCGgatttaaagt
						225071*				++	AACTCGACTCCAtgtccggattggagtctgc
						225100*				- +	aactcgactccatgtccggATTGGAGTCTGC
						225103				++	AATCGCTAGTAAtcgagtcgg
						225192				++	GAAGTAGGTAGCggttgcaaaa
2						225753				++	
						225756*				++	GTACACGGTGGAtgccctggcagttgaggttaagcgactaagc
						**225759**				++	
						225788				- +	cacggtggatgcgttgtgaggttaagCGACTAAGCGTA
						225789*				- +	
						225795				- +	gtacacggtggatgccctggcagttgaggttAAGCGACTAAGC
						**225798***				- +	
						226131*	226130			++	GTATCCTGTCTGatagggcgggacacgtg
						226159*	226158			- +	gtatcctgtctgataggGCGGGACACGTG
								228185		++	AAAAGGTACTCCcggat
								228350		- +	gaacggAGCTGGGTTTA
	23S rRNA frag-ments	*rrlH*	+	225759	228662	226540				++	AAACCGGGAGATagaaggccaatc
						226582				- +	aaagctaccgggagatagcTGGTTCTCCCCG
						227004				++	ATAAGTAACGATaaagcgaagtgcgaatgctgac
						227036				- +	ataagtaacgataaagcgaagtGCGAATGCTGAC
						227148				- +	aaacaggttaaggcgaggccgaaaggcGTAGTCGATGGG
						227285				++	ATGCCCTGCTTCgaggcactacggtgctgaagcaacaa
						227321				- +	atgccctgcttcgaggcactacggtgCTGAAGCAACAA
						227463				- +	aggcaaaaatggtgccgtaACTTCGGGAGA
						227697				++	AGTTCCGACCTGcacgaatggttccttgtcgggta
						227731				- +	agttccgacctgcacgaatggttCCTTGTCGGGTA
						228019				++	AGCACGAAGGTTctcctaaagagtaacggagg
						228050				- +	agcacgaaggttctcctaaaGAGTAACGGAGG

						228087				++	AGCGTGACGGCGtggcataagccagcttgactgcg
						228121				- +	agcgtgacggcggtggcataagccAGCTTGACTGCG
						228316*				++	AGCTGGGTTTAGaccatttaaagtggtacgcg*
						228347**				- +	agctgggtttagaccatttaAAGTGGTACGCG**
						228350***				- +	gaacgGAGCTGGGTTTA***
						228351****				- +	gaacgtGAGCTGGGTTTA****
3		*ylaC*	-	478781	479251				478916	- -	TCTACCGTCAGGgatga
						479165*				+ -	gctttagtatcacgcgACAACAAACCCC
						479191*				- -	GCTTTAGTATCAcgcgacaacaaacccc
4		*ybjJ*	-	886131	887339		886654	886654	886654	+ -	gccttGTGCTGGCGATG
5		*ybjD*	+	916473	918131		919696	919696		- -	TCTCCGTCGCGGagcca
6		*hyfC*	+	2603847	2604794		2604115	2604115		- +	gccttGTGCTGGCGATG
7		*pitA*	+	3637642	3639141		3638344	3638344	3638344	- +	gcattgcgATGGTCAGAAAG
8		*yiaY*	-	3756676	3757827		3747768	3747768		- -	GCGGCTGTCTGAtcagg
9		*creD*	+	4638178	4639530		4638547	4638547		- -	TCGGCTTTTAACcgaaa
